# Aspartic acid-facilitated remineralization: a bio-inspired alternative to fluoride for enamel repair

**DOI:** 10.3389/fbioe.2026.1741728

**Published:** 2026-05-14

**Authors:** Angelina Ivanova, Valeriia Buzova, Diana Potapova

**Affiliations:** 1 SkyLab AG, Superlab Suisse Epalinges SA, Lausanne, Switzerland; 2 School of Physical and Chemical Sciences, Queen Mary University of London, London, United Kingdom

**Keywords:** aspartic acid, calcium sources, enamel remineralization, fluoride, surface microhardness, in vitro

## Abstract

**Background:**

Dental enamel is constantly challenged by acidic conditions that disrupt the balance between demineralization and remineralization. Despite proven efficacy, current remineralization strategies face limitations, which prompt exploration of novel biomimetic approaches. Aspartic acid, a calcium-binding amino acid abundant in enamel matrix proteins, was hypothesized to serve as a potential enhancer of enamel remineralization. This study investigates its potential in combination with calcium sources, comparing the efficacy of these formulations to fluoride as a conventional market benchmark.

**Methods:**

The remineralization potential of the experimental formulations was evaluated using a standardized *in vitro* bovine enamel model quantified by surface microhardness recovery (%SMHR). Polished enamel samples were subjected to demineralization in acidic conditions (pH = 4.5) for 60 min, in a solution containing 50 mM acetic acid, 2.2 mM calcium nitrate, 2.2 mM potassium phosphate monobasic and 0.1 ppm sodium fluoride. After rinsing with deionized water to arrest acid activity, the demineralized samples were incubated with solutions containing aspartic acid, calcium sources, their combinations and fluoride (as positive control) for 16 h at 37 °C. Enamel surface microhardness was measured before and after treatment to assess remineralizing effectiveness of test systems.

**Results:**

While 0.5% aspartic acid alone caused enamel demineralization (mean %SMHR = −37.32 ± 24.64), its combinations with calcium sources outperformed fluoride: 0.5% Asp + 1% tricalcium phosphate system demonstrated a mean %SMHR of 42.03 ± 19.45 - significantly higher than fluoride (14.12% ± 13.40%; p = 0.0181), a system of 0.5% Asp + 1% dicalcium phosphate dihydrate in another experiment achieved a mean %SMHR of 45.43 ± 14.64- also significantly superior to fluoride (5.15% ± 4.84%, p = 0.0058). Other formulations of Asp with calcium sources showed remineralizing potential but lacked statistical superiority to fluoride.

**Conclusion:**

These results suggest that aspartic acid-calcium formulations may offer potential advantages over fluoride-based approaches in promoting enamel remineralization. However, further investigation is needed to elucidate the underlying mechanisms and establish clinical efficacy.

## Graphical Abstract

**Figure:**
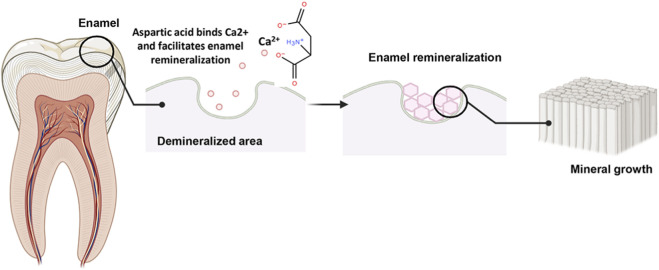
Proposed mechanism of aspartic acid-facilitated enamel remineralization.

## 1 Introduction

Dental enamel, the hardest tissue in the human body (Beniash et al., 2019), is nonetheless susceptible to erosion, which involves degradation and disruption of enamel due to chemical and physical factors. The mineral content of enamel is mostly composed of hydroxyapatite crystals (Lacruz et al., 2017) with reduced calcium content, making it more soluble in acid. Protons can bind to carbonates and phosphates in hydroxyapatite, leaching them from the enamel (Featherstone and Lussi, 2006). In physiological conditions, the balance between demineralization occurring at acidic pH and remineralization at neutral pH is maintained by the mineral depots and buffering capacity of saliva (Farooq and Bugshan, 2021). However, frequent and prolonged pH decrease in the oral cavity significantly increases the risk of enamel erosion by facilitating the dissolution of hydroxyapatite (Lussi et al., 1993; Lechien et al., 2020). Most strategies aim to either stabilize hydroxyapatite and prevent its dissolution or employ compounds that release calcium and phosphate ions into the oral cavity, thereby shifting the balance towards remineralization (Arifa et al., 2019; Lawn et al., 2010; Rodemer et al., 2022).

The strategy, which can be regarded as conventional, involves the use of fluoride formulations. Fluoride ions facilitate the incorporation of calcium (Ca^2+^) and phosphate (PO_4_^3-^) ions into the crystal lattice, leading to the formation of fluorapatite - a compound significantly more resistant to acidic conditions than hydroxyapatite (Ten Cate, 1999). However, these fluorapatite layers form a disordered structure and are considerably different from natural enamel (Yu et al., 2019; Yuwei et al., 2009). Moreover, the effectiveness of fluoride is confined to the enamel surface, with penetration limited to the outer 30 μm of the tooth with minimal impact on deeper layers (Reynolds, 2008; Schmidlin et al., 2016). The stabilization and preservation of natural enamel’s hydroxyapatite can be achieved with arginine (Bijle et al., 2021) and engineered peptides (Marin et al., 2022). These agents prevent demineralization by modulating oral pH levels towards more neutral values. Arginine fosters the growth of beneficial arginolytic bacteria, which produce ammonium as a byproduct of arginine metabolism. This pH increase creates the environment that inhibits proliferation of cariogenic bacteria, thereby promoting oral health and preventing demineralization (Agnello et al., 2017). Although these approaches provide promising strategies for maintaining enamel health, their effectiveness may be limited in advanced stages of demineralization. The third strategy focuses on remineralization that can be facilitated by organic compounds, inorganic soluble compounds, and insoluble sources of calcium and phosphate ions. Organic compounds may act as nucleation sites for enamel remineralization. Сasein phosphopeptide-amorphous calcium phosphate CPP-ACP is one of the most studied non-fluoride remineralizing agents, it has shown significant remineralizing and anticaries effects in several studies (Bailey et al., 2009; Güçlü et al., 2016; Heravi et al., 2019). However, the efficacy of CPP-ACP can vary, as some studies have reported mixed results (Beerens et al., 2010; Bröchner et al., 2011), moreover the substance should be avoided by individuals with allergies to milk proteins. Inorganic soluble compounds, including amorphous calcium phosphate (ACP), provide a readily available source of calcium and phosphate ions for remineralization. However, the limitation of soluble compounds is their potential to contribute to formation of dental calculus (Cochrane et al., 2010). Insoluble sources of calcium and phosphate ions are biocompatible materials that mimic the structure of hydroxyapatite crystals in enamel, nevertheless, they are less effective than soluble compounds due to their slower release of ions (Cochrane et al., 2010; Grohe and Mittler, 2021). Although currently available forms have shown effectiveness in enamel dentistry, each of them presents certain limitations. To enhance the bioavailability and efficacy of calcium sources for remineralization, a biomimetic approach, created by inspiration of natural processes, is essential, and the use of substances that bridge mineral and organic enamel components has been investigated.

Enamel formation occurs during the embryonic period, before tooth eruption, and is regulated by multiple signaling cascades and the activity of ameloblasts, which secrete enamel matrix proteins. Amelogenin constitutes up to 90% of the protein content in developing enamel and plays a key role in directing the hierarchical organization of apatite crystals (Carneiro et al., 2016). Its C-terminus contains a hydrophilic domain rich in acidic amino acid residues, mostly aspartic acid, which can bind to calcium ions and octacalcium phosphate (OCP), an intermediate stage in hydroxyapatite synthesis, thereby guiding hydroxyapatite crystallization (Wu et al., 2017). Due to the negative charge of carboxyl groups, aspartic acid can effectively bind to calcium ions, thereby promoting local supersaturation and serving as a nucleation site (Tavafoghi and Cerruti, 2016). Further, during enamel maturation, the protein content decreases from 35% to 1% (Bartlett, 2013; Smith, 1998), leaving the space for hydroxyapatite crystals (Lu et al., 2008). In adulthood, natural enamel regeneration is impossible due to the absence of cells within the enamel, and remineralization relies solely on calcium and phosphate supply from saliva (Moradian-Oldak, 2009). Modern lifestyle factors and dietary habits create an environment that favors enamel demineralization: consumption of high-sugar and high-acid products causes frequent and significant pH drops that impede enamel’s ability to undergo mineralization (Jayasudha et al., 2014). To address this, the use of aspartic acid was proposed, which, as one of the main amino acid residues of amelogenins (Catalano-Sherman et al., 1993), has been demonstrated to play a key role in natural mineralization processes (Tavafoghi and Cerruti, 2016). Aspartic acid is stable, safe, accessible, and represents a promising biomaterial compound for dental applications. It was shown that 0.5% aspartic acid combined with calcium sources (1% hydroxyapatite) exhibited superior remineralization capacity compared to fluoride-based treatments (Ivanova, 2024). In near-neutral pH environments, the mononegative aspartic acid chelates calcium ions through its carboxylate groups (Liu et al., 2022) and may function as a nucleation site for enamel remineralization, thereby facilitating the synergistic integration of calcium mineral and organic components (Fernando et al., 2022). To enhance the process, calcium sources must have high bioavailability. The primary objective of this research was to investigate the remineralization potential of aspartic acid. Specifically, the study aimed to evaluate the efficacy of aspartic acid in combination with various calcium sources, calcium sources independently and to compare these results against negative control (deionized water) and positive control (fluoride (1,450 ppm F). The null hypothesis proposed that fluoride is the most effective agent for the remineralization of tooth enamel, while the alternative hypothesis suggested that aspartic acid in conjunction with calcium sources could potentially be more effective treatment.

## 2 Materials and methods

### 2.1 Cytotoxicity test. cells and cultivation

#### 2.1.1 Cells and cultivation

Human fibroblasts were obtained from skin of an abdominal plastic surgery patient. Skin tissue biopsy was performed following a written informed consent signed in compliance with the Declaration of Helsinki. The procedure was conducted under the protocol described by Nekrasov et al. (2011). and was approved by the Institutional Review Board of Kazan Federal University (protocol no. 3, 23 March 2017).

The cells were resuspended at a concentration of 1*10^5^ cells per mL in Dulbecco’s Modified Eagle Medium (DMEM, PanEco, Russia) supplemented with 20% fetal bovine serum (FBS, BioSera, France), 2 mM L-glutamine (CAS 56-85-9, PanEco, Russia), 100 μg/mL penicillin (CAS 525-94-0, PanEco, Russia), and 100 μg/mL streptomycin (CAS 57-92-1, PanEco, Russia). They were then cultured in plates in a thermostat at 37 °C in an atmosphere containing 5% CO2 until the formation of monolayer.

#### 2.1.2 MTT assay

Primary fibroblast cultures were seeded into 96-well plates at a density of 1.000–3.000 cells per 200 µL of culture medium, depending on their proliferative potential. After incubation for 24 h in a CO_2_ incubator, the treatment (aspartic acid (CAS 56-84-8/617-45-8, AJINOMOTO, Japan) dilutions at concentrations of 0.156%–10% prepared in DMEM) was given, and the plates were cultured in a CO_2_ incubator (MCO-15AC, Japan) for 48 h. Then the culture medium was removed from the wells and MTT solution (CAS 298-93-1, PanEco, Russia) was added to each well in a volume of 100 µL (the solution for one 96-well plate was prepared by combining 9 mL of culture medium with 1 mL of MTT (3-(4,5-Dimethylthiazol 2-yl)-2,5-diphenyltetrazolium bromide) reagent (5 mg/mL in Hank’s solution (PanEco, Russia)). After the incubation in CO_2_ incubator for 3.5 h, the medium was removed, and formazan crystals formed by the cells were dissolved by adding 100 µL of dimethyl sulfoxide (DMSO, CAS 67-68-5, Ekros, Russia) to each well. The resulting purple coloration was measured at a wavelength of 550 nm using a Tecan Infinite 200 Pro microplate reader (Switzerland). The inhibitory concentration at 50% (IC50), which represents the concentration that is lethal to 50% of cells, was calculated (Sebaugh, 2011). The potential cytotoxic effects of aspartic acid on fibroblasts were further evaluated using light microscopy. Quantification of viable cell areas was performed using BCAnalyzer software (Sinitca et al., 2023).

### 2.2 *In vitro* studies to compare remineralizing abilities of test products

#### 2.2.1 Bovine enamel block preparation

Fifty enamel blocks, measuring 4 *4 mm, were used in the first study, sixty blocks - in the second and one hundred and ten enamel blocks - in the third study. Bovine tooth samples were collected from deceased animals in accordance with ethical guidelines and following approval from the Animal Ethics Committee of Intertek CRS. Blocks were cut from bovine incisors and lapped planar-parallel using a Logitech PM5 lapping machine (United Kingdom). The enamel surfaces of the samples were machine-polished with 1 micron and 0.3 micron aluminium oxide slurries, using a MetPrep Saphir 550 (United Kingdom) to a final finish of 0.04 microns. One corner of each block was removed to ensure correct orientation on the SMH machine. The enamel samples were sonicated, and surface checks performed with an objective lens of the Buehler MicroMet 5,103 microhardness indenter (United States). All prepared blocks were stored in individual Sterilins, between layers of tissue paper dampened with 0.1% thymol.

#### 2.2.2 Surface microhardness measurements (baseline, post-demineralization, post-treatment measurements)

The surface microhardness (SMH) of each enamel block was assessed using a calibrated Buehler MicroMet 5,103 microhardness tester equipped with a Knoop diamond indenter. Enamel samples were positioned with the abraded corner in the top right, and baseline Knoop indents were made at the center of each sample. At each time point, five Knoop indents were made in a vertical line, applying a load of 50 g for 10 s. To qualify for further analysis, each enamel block had to achieve an average baseline SMH greater than 250.0 HK and a standard deviation of less than 20.0. Post-demineralization and post-treatment SMH measurements were conducted approximately 100 microns from the baseline indents, with five Knoop indents placed vertically under the same loading conditions (50 g for 10 s). The initial surface microhardness (SMH) of the enamel samples was measured to establish baseline values. Based on these values, the enamel blocks were stratified between the treatment groups to ensure similar mean baseline SMH values in each group (n = 10). Specimens with “outlier” baseline SMH values were removed from the study. Subsequently, blocks were subjected to demineralization by immersion in a demineralizing solution. After demineralization, the surface microhardness of the samples was reassessed.

#### 2.2.3 Enamel block demineralization

Polished enamel samples were subjected to demineralization in acidic conditions for 60 min, in a solution containing 50 mM acetic acid, 2.2 mM calcium nitrate, 2.2 mM potassium phosphate monobasic, 0.1 ppm sodium fluoride. Final pH of the solution was adjusted to 4.5 with sodium hydroxide (Huang et al., 2009). Enamel blocks were fixed on acetate squares, each sample was immersed in 8 mL of demineralizing solution at 37 °C. After an hour of incubation, the samples were removed from the solution, rinsed with deionized water for 2 min, and returned to their containers.

#### 2.2.4 Studies to compare remineralizing abilities of test products

Individual ingredients used for preparation of test solutions are described in Table 1.

**TABLE 1:** Individual ingredients used in experiments.

№	Ingredient	CAS	Supplier
1	Aspartic acid (Asp)	56-84-8/617-45-8	AJINOMOTO, Japan
2	Sodium fluoride (F)	7681-49-4	Sigma-aldrich, United Kingdom
3	CaMgZnHAP (CMZ)	​	Sigma-aldrich, United Kingdom
4	Tricalcium phosphate (TCP)	7758-87-4	Sigma-aldrich, United Kingdom
5	nanoXim®•CarePaste Hydroxyapatite (nano) (nanoXim)	​	Fluidinova S.A., Portugal
6	Deionized water (DW)	7732-18-5	​
7	Sodium monofluorophosphate (MF)	7789-74-4	Wendeng jinye industrial Co., Ltd., China
8	Dicalcium phosphate dihydrate (DPD)	7789-77-7	Reephos (Chongqing) food ingredients Co., Ltd., China

Treatment products and preparation of test solutions are presented in Table 2. Enamel samples in each treatment group (n = 10) were attached to a modified sample holder using Aquasil Hard Putty (United States) and treated with the assigned test product for 16 h. All stages of treatment were carried out in an incubator at 37 °C using a magnetic stirrer. A duration of 16 h was selected to ensure a distinct differentiation in the results among the various treatment groups. Following the removal of the samples from the test product, each enamel block was rinsed with deionized water for 2 min and sonicated for 10 min to remove any loose debris. The enamel samples were then returned to their original containers.

**TABLE 2:** Treatment products used in experiments.

Treatment	Preparation of test solutions
1,450 ppm F (positive control)	0.3238 g of NaF powder made up to 100.04 g with deionised water. pH of the solution was 7.17 (not adjusted)
Deionized water (negative control)	Used as supplied by manufacturer
1% CaMgZnHAp	0.41 g CaMgZnHAP dry powder was dissolved in deionized water to achieve a total mass of 40.32 g. 7.07 pH was adjusted by 1% HCl
1% tricalcium phosphate	0.40 g tricalcium phosphate dry powder was dissolved in deionized water (total mass is 40.15 g), pH of the solution was 7.02 (not adjusted)
nanoXim®•CarePaste Hydroxyapatite (nano)	2.70 g nanoXim®•CarePaste hydroxyapatite (nano) 15% solution made up to 40.01 g with deionised water, pH adjusted to 7.17 by 1% HCl
Artificial saliva	400 mg sodium Chloride (NaCl), 1,210 mg potassium Chloride (KCl), 780 mg sodium phosphate monobasic dihydrate (NaH2PO4.2H2O), 5 mg sodium sulphide nonahydrate (Na2S.9H2O), 1,000 mg Urea (CO(NH2)2), 1,000 mL deionised water
0.05% aspartic acid	0.020 g of aspartic acid made up to 40 g with deionised water
0.2% aspartic acid	0.080 g of aspartic acid made up to 40 g with deionised water
0.2% aspartic acid in artificial saliva	0.080 g of aspartic acid made up to 40 g with artificial saliva
0.4% aspartic acid	0.160 g of aspartic acid made up to 40 g with deionised water
0.5% aspartic acid + 1% CAMgZnHAP	0.200 g of aspartic acid, 0.400 g of CaMgZnHAP made up to 40 g with deionised water
0.5% aspartic acid + 1% nanoXim®•CarePaste hydroxyapatite (nano)	0.200 g of aspartic acid, 3.000 g of nanoXim®•CarePaste hydroxyapatite (nano) made up to 40 g with deionised water
1% aspartic acid +1.5% tricalcium phosphate	0.400 g of aspartic acid, 0.600 g ot Tricalciup Phosphate made up to 40 g with deionised water
0.5% aspartic acid + monofluorophosphate 1,450 ppm	0.200 g of aspartic acid, 0.4395 g of sodium monofluorophosphate made up to 40 g with deionised water
0.5% aspartic acid + 1% dicalcium phosphate dihydrate	0.200 g of aspartic acid, 0.400 g of dicalcium phosphate dihydrate made up to 40 g with deionised water
0.1% aspartic acid	0.040 g of aspartic acid made up to 40 g with deionised water
0.1% aspartic acid + 0.5t Tricalciup Phosphate	0.040 g of aspartic acid, 0.200 g ot Tricalciup Phosphate made up to 40 g with deionised water
0.5% aspartic acid + 1% tricalcium phosphate	0.200 g of aspartic acid, 0.400 g ot Tricalciup Phosphate made up to 40 g with deionised water

The pH of each solution was adjusted into the range of 6–6.5, using sodium hydroxide or diluted hydrochloric acid (excluding positive and negative controls). All pH measurements were taken with a calibrated pH electrode (Serial Number: VT1 8048) and an Accumet XL250 ion meter (Asset Number: UK01200-LAB-15-002).

### 2.3 Data management

#### 2.3.1 Statistical analysis

Cytotoxicity experiments were carried out in triplicates. Statistical analysis was conducted using GraphPad Prism 8.4.3. (GraphPad SoftwareInc, 2020), along with BioFilmAnalyzer (Bogachev et al., 2018) and BCAnalyzer software.

The remineralization potential was quantified by calculating the percentage recovery of surface microhardness (%SMHR) using the following formula:

$$\%\text{SMHR}=100\times \frac{\left(\text{SMH}\;\text{post}\;\text{treatment}-\text{SMH}\;\text{post}\;\text{demineralization}\right)}{\left(\text{SMH}\;\text{baseline}-\text{SMH}\;\text{post}\;\text{demineralization}\right)}$$

Minitab18 software (Minitab, 2017) was used to calculate mean, median, maximum, minimum and standard deviation of %SMHR for each test product. Statistical analysis was performed using GraphPad Prism 8.4.3. The %SMHR values for enamel lesions treated by the test products was statistically compared using a General Linear Model ANOVA. A Tukey test was selected to make pairwise statistical comparisons.

#### 2.3.2 Sample size calculation

Sample size determination was conducted using G*Power 3.1.9.7 (Faul et al., 2007) with Cohen’s effect size f = 0.60, significance level α = 0.05, statistical power 0.85, and one-way ANOVA design. This effect size was selected based on preliminary observations and literature analysis indicating substantial mean %SMHR differences between treatment groups in highly controlled *in vitro* conditions (Sahiti et al., 2020). Moreover, the 16-h treatment duration was selected to allow sufficient time for remineralization or demineralization processes and to ensure a distinct differentiation in the results. *A priori* power analysis indicated minimum sample sizes of n = 10, 8, and 5-6 per group for Experiments 1, 2, and 3, respectively. However, we selected n = 10 per group across all three experiments for the following reasons: 1- to maintain consistent sample size across experiments and enhance comparability of effect sizes; 2- to provide robustness against potential data exclusion; and 3- to increase precision of effect size estimation and confidence intervals.

#### 2.3.3 Comparison of data across experiments

Given the variability in the %SMHR values of positive and negative controls (1,450 ppm fluoride and deionized water, respectively) across independent experiments, a direct comparison of %SMHR values was not possible. Consequently, all mean %SMHR values achieved by treatment groups were normalized into standardized “units”.

The calculation was conducted as follows:

For each individual experiment (1-3), the absolute difference between the mean %SMHR values of positive control and negative control was calculated and defined as 100 units (representing the efficacy range of fluoride relative to water within each experiment). For treatment groups exhibiting %SMHR values exceeding that of the positive control, the magnitude of the difference between the test group and the fluoride was determined. This value was then converted into units based on the experiment-specific reference scale. For treatment groups exhibiting %SMHR values lower than that of the positive control, performance was quantified by calculating the differential relative to the negative control and converting this value into units. While this standardization provides relative rather than absolute values, it enables the direct ranking of remineralization effectiveness across independent experiments.

## 3 Results

### 3.1 Cytotoxicity assessment of aspartic acid

In MTT assay for primary fibroblast culture, the IC50 value of aspartic acid was found to be 5.3%. The potential cytotoxic effects of aspartic acid on fibroblasts were further evaluated using light microscopy and quantification of viable cell areas was performed using BCAnalyzer software. Notably, at the highest concentration tested (2.5% aspartic acid), viable cells were still detected.

### 3.2 In vitro studies to compare remineralizing abilities of test products

#### 3.2.1 Remineralizing efficacy of various calcium sources

The first experiment was aimed at determining the remineralization effectiveness of different calcium sources. The following products were used: 1% nanoXim®•CarePaste Hydroxyapatite (nano), CaMgZnHAP and tricalcium phosphate. Surface microhardness recovery (%SMHR) values were used as a metric for remineralization potential of test products and were compared to positive and negative controls-fluoride and deionized water, respectively. The results are shown in Table 3.

**TABLE 3:** Statistical comparison of remineralization effectiveness: post-treatment %SMHR values and P-value ranking of tested calcium sources.

%SMHR						
Treatment	​	F	1% CMZ	1% nanoXim	1% TCP	DW
​	Mean ± SD	35.56 ± 23.41	26. 68 ± 25.30	21.34 ± 14.31	14.50 ± 14.23	−1.87 ± 17.11
1,450 ppm F	35.56 ± 23.41	​	0.8443	0.4835	0.1278	**0.0008**
1% CaMgZnHAP	26. 68 ± 25.30	0.8443	​	0.9721	0.6297	**0.0161**
1% nanoXim	21.34 ± 14.31	0.4835	0.9721	​	0.9328	0.0745
1% tricalcium phosphate	14.50 ± 14.23	0.1278	0.6297	0.9328	​	0.3411
Deionized water	−1.87 ± 17.11	**0.0008**	**0.0161**	0.0745	0.3411	​

The %SMHR, values were statistically compared using a General Linear Model ANOVA. A Tukey test was selected to make pairwise statistical comparisons. **Bold** adjusted p-values indicate statistically significant differences between the %SMHR, values achieved be the different treatments.

For clarity of presentation, treatment systems were organized by %SMHR values and grouped according to statistical significance using a multiple comparisons test (Table 4). Statistical significance between groups is indicated by letters (means that do not share a letter are significantly different).

**TABLE 4:** Statistical grouping of treatment systems by achieved %SMHR values in multiple comparisons test.

Treatment	%SMHR	StDev	Grouping			N
1,450 ppm F	35.56	23.41	A	​	​	10
1% CaMgZnHAP	26. 68	25.30	A	B	​	10
1% nanoXim	21.34	14.31	A	B	C	10
1% TCP	14.50	14.23	A	B	C	10
Deionized water	−1.87	17.11	​	​	C	10

%SMHR-Percentage surface microhardness recoveries.

**FIGURE 1:**
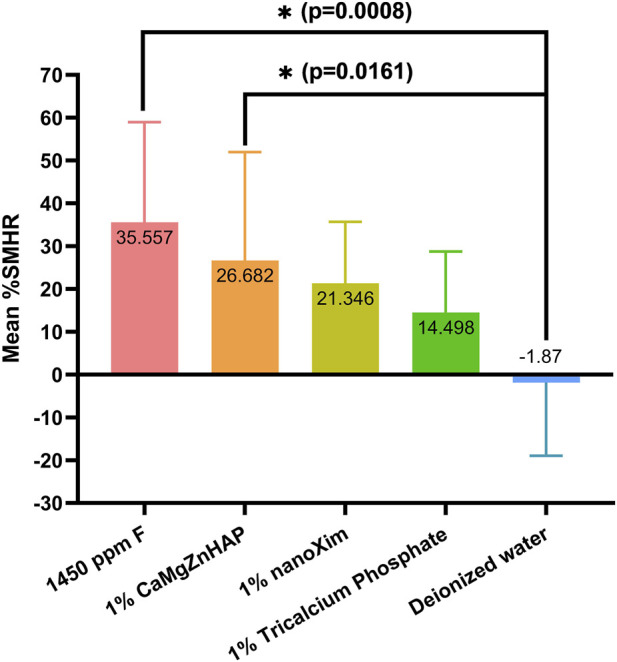
Surface microhardness recoveries (%SMHR) of demineralized enamel samples treated with various calcium sources, fluoride and deionized water. Data are presented as mean ± standard deviation.

The results indicate that the fluoride exhibited the highest remineralizing effectiveness among the tested products, achieving an average %SMHR of 36% (35.56 ± 23.41), followed by 1% CaMgZnHAP, (26.68 ± 25.30), 1% nanoXim (21.34 ± 14.31) and 1% TCP (14.50 ± 14.23). Deionized water caused slight demineralization, resulting in a mean %SMHR value of −2% (−1.87 ± 17.11). Among the tested treatment systems, only fluoride (p = 0.0008) and 1% CMZ (p = 0.0161) induced statistically significant remineralization relative to the negative control. Results are graphically summarized in Figure 1.

#### 3.2.2 Remineralizing efficacy of aspartic acid at varying concentrations and in combination with tricalcium phosphate

This study assessed the remineralization effectiveness of aspartic acid when applied individually and in combination with TCP, with particular emphasis on investigating the concentration-dependent effects. The following solutions were applied to enamel samples: 0.1% Asp, 0.5% Asp, 0.1% Asp +0.5% TCP, 0.5% Asp + 1% TCP; fluoride and deionized water were used as positive and negative controls, respectively. The results are presented in Table 5.

**TABLE 5:** P-value ranking of test products by differences in Post-Treatment mean %SMHR in the second experiment.

	%SMHR						​
Treatment	​	0.5% Asp + 1% TCP	0.1% Asp +0.5% TCP	1,450 ppm F	0.1% Asp	DW	0.5% Asp
	↓Mean ± SD→	42.03 ± 19.45	32.13 ± 18.85	14.12 ± 13.40	3.19 ± 9.37	−17.52 ± 23.54	−37.32 ± 24.64
0.5% Asp + 1%TCP	42.03 ± 19.45	​	0.8422	**0.0181**	**0.0003**	**<0.0001**	**<0.0001**
0.1% Asp + 0.5% TCP	32.13 ± 18.85	0.8422	​	0.2757	**0.0128**	**<0.0001**	**<0.0001**
1,450 ppm F	14.12 ± 13.40	**0.0181**	0.2757	​	0.7798	**0.0049**	**<0.0001**
0.1% Asp	3.19 ± 9.37	**0.0003**	**0.0128**	0.7798	​	0.1487	**0.0002**
Deionized water	−17.52 ± 23.54	**<0.0001**	**<0.0001**	**0.0049**	0.1487	​	0.1856
0.5% Asp	−37.32 ± 24.64	**<0.0001**	**<0.0001**	**<0.0001**	**0.0002**	0.1856	​

The %SMHR, values were statistically compared using a General Linear Model ANOVA. A Tukey test was selected to make pairwise statistical comparisons. Bold adjusted p-values indicate statistically significant differences between the %SMHR, values achieved be the different treatments.

The treatment systems were ranked by their %SMHR values in Table 6. Statistical significance in pairwise comparisons is represented by letters: means sharing a letter are not statistically different, whereas those with different letters exhibit significant differences.

**TABLE 6:** Post-treatment mean %SMHR values, standard deviations, and statistical groupings for each treatment in the experiment evaluating the remineralizing efficacy of aspartic acid and its combinations with tricalcium phosphate.

Treatment	Mean %SMHR	StDev	Grouping					N
0.5% Asp + 1% TCP	42.03	19.45	A	​	​	​	​	10
0.1% Asp +0.5% TCP	32.13	18.85	A	B	​	​	​	10
1,450 ppm F	14.12	13.40	​	B	C	​	​	10
0.1% Asp	3.19	9.37	​	​	C	D	​	10
Deionized water	−17.52	23.54	​	​	​	D	E	10
0.5% Asp	−37.32	24.64	​	​	​	​	E	10

%SMHR-Percentage surface microhardness recoveries; StDev, Standard Deviation.

**FIGURE 2:**
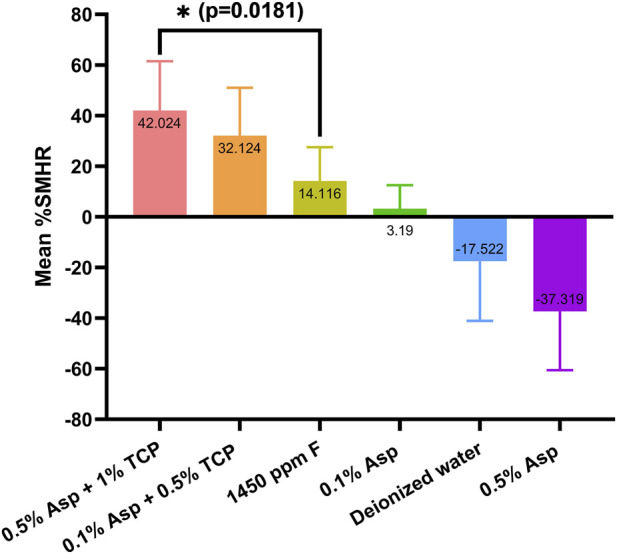
Surface microhardness recoveries (%SMHR) of demineralized enamel samples treated with aspartic acid and its combinations with TCP. Data are presented as mean ± standard deviation.

Following a single overnight application, the 0.5% aspartic acid + 1% TCP system demonstrated the highest remineralizing efficacy, achieving a mean %SMHR of 42.03% ± 19.45% - significantly greater than that of the 1,450 ppm fluoride (14.12% ± 13.40%; p = 0.0181). The system with lower concentration of Asp and TCP (0.1% Asp +0.5% TCP) also exhibited considerable remineralization (32.13% ± 18.85%), however it was not statistically superior to fluoride (p = 0.2757). The remineralizing efficacy of aspartic acid was evaluated at two concentrations: 0.1% and 0.5%. At 0.1%, aspartic acid achieved a mean %SMHR of 3.19 ± 9.37, showing no significant difference from either the positive control (p = 0.7798) or the negative control (p = 0.1487), although a slight remineralization trend was observed. In contrast, 0.5% Asp induced substantial demineralization (mean %SMHR = −37.32% ± 24.64%), exceeding the negative control (mean %SMHR = −17.52% ± 23.54%). However, this difference was not statistically significant (p = 0.1856). Results are graphically summarized in Figure 2.

#### 3.2.3 Assessment of remineralizing potential of aspartic acid combined with various calcium sources

In the subsequent study, the remineralizing efficacy of 0.5% aspartic acid (previously tested individually) was assessed in combinations with various calcium sources. Additionally, the concentration-dependent effects of Asp at 0.05%, 0.2%, and 0.4% were investigated. The following solutions were applied to enamel samples: 0.5% Asp + 1% DPD, 0.5% Asp + 1% CMZ, 0.5% Asp + 1% nanoXim, 1% Asp + 1.5% TCP, 0.5% Asp + Monofluorophosphate, 0.05% Asp, 0.2% Asp, 0.4% Asp; mean %SMHR values were measured for each solution and compared to %SMHR values of positive and negative controls. The results are presented in Table 7.

**TABLE 7:** Statistical comparison of remineralization effectiveness: post-treatment %SMHR values and P-value ranking of tested systems.

%SMHR											
Treatment	​	0.5% Asp + 1% DPD	0.5% Asp + 1% CMZ	0.5% Asp + 1% nanoXim	1% Asp +1.5% TCP	1,450 ppm F	0.5% Asp + MF	DW	0.05% Asp	0.2% Asp	0.4% Asp
​	Mean ± SD	45.43 ± 14.64	33.08 ± 14.84	26.16 ± 13.91	22.54 ± 8.65	5.15 ± 4.84	−11.07± 11.16	−29.21± 18.38	−35.13 ± 24.84	−87.69± 47.76	−91.74± 27.01
0.5% Asp + 1% DPD	45.43 ± 14.64	​	0.9688	0.6768	0.4359	**0.0058**	**<0.0001**	**<0.0001**	**<0.0001**	**<0.0001**	**<0.0001**
0.5% Asp + 1% CAMgZnHAP	33.08 ± 14.84	0.9688	​	0.9996	0.9894	0.1756	**0.0016**	**<0.0001**	**<0.0001**	**<0.0001**	**<0.0001**
0.5% Asp + 1% nanoXim	26.16 ± 13.91	0.6768	0.9996	​	>0.9999	0.5611	**0.0153**	**<0.0001**	**<0.0001**	**<0.0001**	**<0.0001**
1% Asp +1.5% TCP	22.54 ± 8.65	0.4359	0.9894	>0.9999	​	0.7906	**0.0438**	**<0.0001**	**<0.0001**	**<0.0001**	**<0.0001**
1,450 ppm F	5.15 ± 4.84	**0.0058**	0.1756	0.5611	0.7906	​	0.8500	**0.0355**	**0.0058**	**<0.0001**	**<0.0001**
0.5% Asp + MF	−11.07 ± 11.16	**<0.0001**	**0.0016**	**0.0153**	**0.0438**	0.8500	​	0.7472	0.3640	**<0.0001**	**<0.0001**
Deionized water	−29.21 ± 18.38	**<0.0001**	**<0.0001**	**<0.0001**	**<0.0001**	**0.0355**	0.7472	​	0.9999	**<0.0001**	**<0.0001**
0.05% Asp	−35.13 ± 24.84	**<0.0001**	**<0.0001**	**<0.0001**	**<0.0001**	**0.0058**	0.3640	0.9999	​	**<0.0001**	**<0.0001**
0.2% Asp	−87.69 ± 47.76	**<0.0001**	**<0.0001**	**<0.0001**	**<0.0001**	**<0.0001**	**<0.0001**	**<0.0001**	**<0.0001**	​	>0.9999
0.4% Asp	−91.74 ± 27.01	**<0.0001**	**<0.0001**	**<0.0001**	**<0.0001**	**<0.0001**	**<0.0001**	**<0.0001**	**<0.0001**	>0.9999	​

The %SMHR, values were statistically compared using a General Linear Model ANOVA. A Tukey test was selected to make pairwise statistical comparisons. **Bold** adjusted p-values indicate statistically significant differences between the %SMHR, values achieved be the different treatments.

The treatment systems were ranked by their %SMHR, values in Table 8. Statistical significance between groups is indicated by letters (means that do not share a letter are significantly different).

**TABLE 8:** Post-treatment mean %SMHR values, standard deviations, and statistical groupings for each treatment in the experiment evaluating the remineralizing efficacy of aspartic acid and its combinations with various calcium sources.

Treatment	Mean %SMHR	StDev	Grouping					N
0.5% Asp + 1% DPD	45.43	14.64	A	​	​	​	​	10
0.5% Asp + 1% CAMgZnHAP	33.08	14.84	A	B	​	​	​	10
0.5% aspartic acid + 1% nanoXim	26.16	13.91	A	B	​	​	​	10
1% aspartic acid + 1.5% TCP	22.54	8.65	A	B	​	​	​	10
1,450 ppm F	5.15	4.84	​	B	C	​	​	10
0.5% Asp + Monofluorophosphate	−11.07	11.16	​	​	C	D	​	10
Deionized water	−29.21	18.38	​	​	​	D	​	10
0.05% Asp	−35.13	24.84	​	​	​	D	​	10
0.2% Asp	−87.69	47.76	​	​	​	​	E	10
0.4% Asp	−91.74	27.01	​	​	​	​	E	10

%SMHR-Percentage surface microhardness recoveries; StDev- Standard Deviation.

**FIGURE 3:**
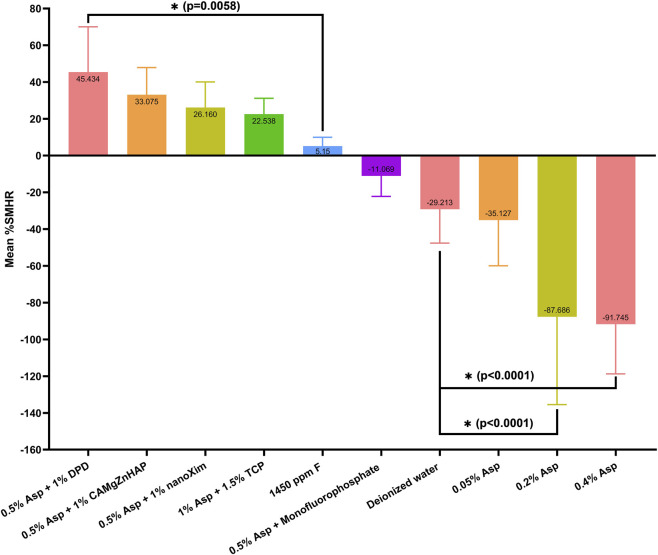
Mean percentage surface microhardness recoveries (%SMHR) of demineralized enamel samples treated with aspartic acid in different concentrations and with various calcium sources, compared to positive and negative controls. Data are presented as mean ± standard deviation.

Following a single overnight application, the system containing 0.5% Asp and 1% DPD exhibited the highest remineralizing efficacy, achieving a mean %SMHR of 45% (45.43 ± 14.64), which is significantly higher (p = 0.0058) than fluoride’s mean %SMHR value (5.15 ± 4.84). Other Asp-calcium combinations also exhibited substantial remineralization, including 0.5% Asp + 1% CMZ (33.08% ± 14.84%; p = 0.1756) and 0.5% Asp + 1% nanoXim (26.16% ± 13.91%; p = 0.5611), although neither was statistically superior to fluoride. Notably, the solution with higher concentrations of Asp + TCP (1% and 1.5%, respectively) underperformed relative to other Asp-calcium combinations (22.54% ± 8.65%; p = 0.7906), indicating that elevated concentrations of aspartic acid do not enhance remineralization. Unexpectedly, the 0.5% Asp + MF system induced demineralization with a mean %SMHR of −11.07% ± 11.16% (p = 0.7472 compared with %SMHR values of DW, negative control, −29.21 ± 18.38). Asp individually produced progressively severe demineralization: 0.05% Asp (−35.13% ± 24.84%; p = 0.9999 vs. negative control), 0.2% Asp (−87.69% ± 47.76%; p < 0.0001), and 0.4% Asp (−91.74% ± 27.01%; p < 0.0001). Results are graphically summarized in Figure 3.

As a supplementary experiment, the remineralizing efficacy of 0.2% aspartic acid in artificial saliva was evaluated. This group was excluded from statistical comparison table due to its fundamentally different physicochemical environment, characterized by high ionic strength and mineral supersaturation, compared to the deionized water used in other experimental groups. Notably, 0.2% aspartic acid shifted from severe demineralization in deionized water (mean %SMHR = −87.69 ± 47.76) to a remineralization in artificial saliva (mean %SMHR = 16.21 ± 8.35).

**FIGURE 4:**
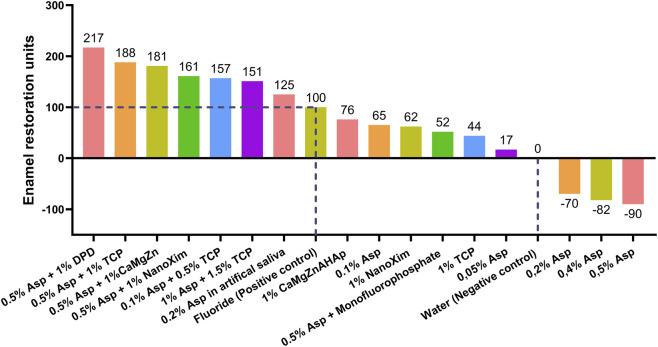
Relative enamel restoration efficacy of test groups across three experiments (standardized units).

To compare data across experiments 1, 2, and 3, results were combined into a single unified table (Table 9; Figure 4). Given the variability in the %SMHR values of the positive and negative controls across separate experiments, a direct comparison of %SMHR values was not possible. Consequently, all mean %SMHR values were normalized into standardized “units”. In each experiment, the difference between the positive control (fluoride) and the negative control (deionized water) was set as 100 units. For treatments outperforming fluoride, the difference above the positive control was calculated and converted into units, for treatments performing below fluoride, remineralizing effectiveness was measured relative to the negative control.

**TABLE 9:** Comparative efficacy of different formulations across three independent experiments, normalized to a standardized scale where the efficacy gap between positive (fluoride) and negative (water) controls equals 100 units.

System	Comparison
0.5% Asp + 1% DPD	For 117 units better than positive control
0.5% Asp + 1% TCP	For 88 units better than positive control
0.5% Asp + 1% CaMgZnHAp	For 81 units better than positive control
0.5% Asp + 1% NanoXim	For 61 units better than positive control
0.1% Asp + 0.5% TCP	For 57 units better than positive control
1% Asp + 1.5% TCP	For 51 units better than positive control
0.2% Asp in artifical saliva	For 25 units better than positive control

Fluoride (positive control)	100 units
1% CaMgZnAHAp	For 76 units better than negative control
0.1% aspartic acid	For 65 units better than negative control
1% NanoXim	For 62 units better than negative control
0.5% aspartic acid + monofluorophosphate	For 52 units better than negative control
1% TCP	For 44 units better than negative control
0.05% Asp	For 17 units better than negative control

Water (negative control)	0 units
0.2% Asp	For 70 units worse than negative control
0.4% Asp	For 82 units worse than negative control
0.5% Asp	For 90 units worse than negative control

## 4 Discussion

In the initial phase of the study investigating the remineralization potential of individual calcium sources, the 1% CaMgZnHAP demonstrated statistically significant enamel repair compared to the negative control (deionized water), although fluoride exhibited superior efficacy. Building upon these findings, the subsequent investigation focused on aspartic acid, a key monomer of enamel matrix proteins (Wu et al., 2017), to elucidate its potential as a biomimetic facilitator of remineralization. The experimental data revealed a distinct concentration-dependent duality in Asp effects: while higher concentrations (0.2%, 0.4%, and 0.5%) induced pronounced demineralization, lower concentrations (0.1% and 0.05%) exhibited a trend towards remineralization. This bimodal behavior can be attributed to the specific physicochemical competition between surface adsorption kinetics and solution thermodynamics described in recent literature (Tavafoghi and Cerruti, 2016). At neutral and slightly acidic pH in solution, Asp possesses two negatively charged carboxyl groups, capable of chelating calcium ions. When supplemental calcium is available, this chelation directs hydroxyapatite mineralization, whereas excess aspartic acid binds to hydroxyapatite surface and becomes a chelator inducing dissolution (Tavafoghi and Cerruti, 2016). Consequently, precise control over the aspartic acid to calcium ratio is crucial for achieving synergistic effects on remineralization.

There were also differences observed in the effects of Asp in deionized water and artificial saliva, despite both solutions were adjusted to a safe physiological pH of 6.0–6.5. The widespread belief that enamel dissolution occurs only below a “critical pH” of 5.5 is valid when the surrounding fluid contains calcium and phosphate ions typical of saliva. In deionized water, which represents undersaturated environment, >0.1% Asp functions as a calcium chelator, and enhances dissolution, driven by the lack of ionic saturation. In contrast, aspartic acid in saliva acts as a facilitator of nucleation, recruiting calcium ions to repair the hydroxyapatite lattice. Chemical erosion of enamel occurs either by hydrogen ions derived from acids, or by chelators binding and forming complexes with calcium (Featherstone and Lussi, 2006). Distilled water represents a non-physiological condition, devoid of the supersaturation that normally prevents demineralization. Calcium ions released from the enamel surface through ion exchange are sequestered by charged carboxyl groups of Asp. Moreover there is no precipitation of calcium-phosphate phases in the absence of phosphate ions, and aspartic acid has no competitors for calcium chelation. The result is progressive demineralization, manifested by the substantial negative %SMHR value observed in the study. The composition of the artificial saliva creates a fundamentally different chemical context. At pH 6-6.5 in a phosphate-buffered system, any dissolved calcium and phosphate ions exist in a state of supersaturation, creating a driving force for mineralization. Moreover, the presence of phosphates in artificial saliva alters aspartic acid’s chelating ability-phosphate ions compete with Asp for calcium coordination, reinforcing the precipitation of calcium-phosphates. Thus, aspartic acid in a buffered system and in the presence of phosphate ions undergoes a functional transition from inhibitor to facilitator of mineralization. The carboxyl groups of aspartic acid interact with the precursor phases, promoting the oriented transformation into hydroxyapatite crystals (Zhao et al., 2021). Our findings of Asp effects in artificial saliva demonstrate its safety for oral applications even in formulations lacking calcium sources, since native saliva functions as a physiological buffering system, abundant with calcium and phosphate ions. It also should be noted that these proposed mechanisms of Asp effects are hypothesis-driven and require further experimental validation.

After assessing calcium sources and Asp individually, effects of their combinations were evaluated. Formulations of 0.5% aspartic acid + 1% DCPD and 0.5% aspartic acid + 1% TCP demonstrated significantly higher %SMHR values than fluoride. Other combinations of Asp with calcium sources (CaMgZnHAP, nanoXim) showed remineralizing potential but lacked statistical significance. The superior efficacy of 0.5% Asp with 1% DCPD or 1%TCP compared to 1% CaMgZnHAP and 1% nanoXim reflects differences in the solubility of these calcium phosphate phases. Unlike crystalline hydroxyapatites, DCPD (CaHPO_4_·2H_2_O) and TCP (Ca_3_(PO_4_)_2_) are thermodynamically unstable precursor phases that dissolve and re-precipitate in solution, providing free Ca^2+^ and PO_4_^3-^ ions. DCPD’s highest solubility explains its superior performance; TCP, with intermediate solubility, however, provides more controlled and consistent ion release (Meyer et al., 2018; Kalra et al., 2014).

In contrast, nanoXim and CaMgZnHAP are stable hydroxyapatite phases with minimal solubility at physiological pH, so they can promote remineralization by the deposition of HAP particles on tooth surfaces and forming mineral-mineral bridges with enamel crystals (Meyer et al., 2022). CaMgZnHAP is a magnesium and zinc-doped hydroxyapatite. The incorporation of Zn^2+^ and Mg^2+^ into the hydroxyapatite induces structural defects and decreases overall crystallinity. These crystalline defects increase solubility relative to intact hydroxyapatite (Dornelas et al., 2024; Garbo et al., 2020), resulting in modest calcium ion release at physiological pH. Despite this theoretical advantage for aspartic acid coordination, the capacity of ion release remains substantially lower than that of DCPD or TCP, and may be further limited by competition between Mg^2+^ and Zn^2+^ ions for coordination with aspartic acid carboxyl groups. NanoXim, consisting of pure hydroxyapatite nanoparticles, exhibits high thermodynamic stability (Sun et al., 2024), although the reduced particle size provides increased specific surface area, which theoretically enhances interactions with aspartic acid.

The combination of 0.5% aspartic acid with monofluorophosphate failed to demonstrate synergy and resulted in enamel demineralization. Monofluorophosphate releases negatively charged ions (F^−^ and PO_4_^3-^), unlike DCPD or TCP, which supply Ca^2+^ ions, and cannot satisfy aspartic acid’s chelation demand. In the absence of exogenous calcium sources, Asp binds directly to calcium from enamel hydroxyapatite, promoting dissolution rather than remineralization (Tavafoghi and Cerruti, 2016). Subsequently, Asp and MF in deionized water may perform as competitors, depleteng enamel calcium. However, the exact mechanism remains to be elucidated through additional studies.

The results indicate that aspartic acid exhibits concentration-dependent effects on enamel: although high concentrations of Asp can demineralize enamel, its combinations with calcium sources demonstrate synergistic remineralizing efficacy. The underlying molecular mechanisms governing its coordination with calcium sources and subsequent hydroxyapatite nucleation need deeper investigation. Specifically, characterizing the distinction between calcium-coordinated aspartic acid that promotes crystal growth and free, uncoordinated aspartic acid that inhibits crystallization.

Significant limitations of the study include assessment via surface microhardness alone. While SMH has been shown to correlate with mineral content changes in enamel (Lippert and Lynch, 2014), it is still an indirect measure and does not provide quantitative information on mineral density, and the distribution of mineral recovery. Complementary techniques such as transverse microradiography or micro-computed tomography could be incorporated to enable more detailed characterization of mineral density and architecture.

Regarding the cytotoxicity assessment, the use of a fibroblast model represents a simplified approach that does not fully replicate the barrier properties of the oral epithelium. Future studies employing primary oral keratinocytes or 3D mucosal models would provide a more tissue-relevant assessment.

In addition, further work is needed to define optimal Asp-calcium ratios, provide clinical validation of *in vitro* findings, and employ secondary erosive challenge to assess whether remineralized enamel maintains protective capacity against acid exposure.

However, our study establishes aspartic acid combinations with calcium sources as a promising strategy for enamel remineralization and provides a foundation for subsequent research. Beyond efficacy, aspartic acid offers two critical advantages for oral care products: cost-effectiveness and safety in pediatric applications-unlike fluoride, it does not risk dental fluorosis during the critical enamel maturation period.

## 5 Conclusion

The results indicate that aspartic acid exhibits concentration-dependent effects on enamel: although high concentrations of aspartic acid can demineralize enamel, its combination with calcium sources may offer potential advantages over fluoride-based approaches in promoting enamel remineralization. However, these findings apply to controlled *in vitro* conditions, further investigation is needed to elucidate the underlying mechanisms and establish clinical efficacy.

## Data Availability

The raw data are available in the supplementary material.
