# Supplementary material for: Aspartic acid-facilitated remineralization: a bio-inspired alternative to fluoride for enamel repair
Source: Front Bioeng Biotechnol. 2026 May 14;14:1741728. doi: 10.3389/fbioe.2026.1741728 (PMC13216666; doi:10.3389/fbioe.2026.1741728)
Supplement: Supplementary file 1 [file DataSheet1.pdf]

Table S1. First experiment surface microhardness raw data (assessment of remineralizing efficacy of various calcium sources).

Treatment Group	Stage	HK1	HK2	HK3	HK4	HK5	Average	StnDev
**1450 ppm F**	Baseline	355	351	355	330	348	347.75	11.98
	Post-Demineralizationeralization	301	300	307	311	302	304.20	4.66
	Post-Treatmentmentment	352	333	351	325	348	341.80	12.11
	Baseline	308	306	322	324	295	311.00	12.04
	Post-Demineralizationeralization	233	171	228	195	187	202.80	26.78
	Post-Treatmentment	239	254	214	201	233	228.20	20.90
	Baseline	237	246	260	277	263	256.60	15.53
	Post-Demineralizationeralization	176	216	194	213	195	198.80	16.24
	Post-Treatmentment	226	226	238	220	229	227.80	6.57
	Baseline	358	372	342	332	346	350.00	15.43
	Post-Demineralizationeralization	206	247	222	220	244	227.80	17.33
	Post-Treatmentment	233	271	248	243	266	252.20	15.93
	Baseline	329	324	333	342	317	329.00	9.41
	Post-Demineralizationeralization	244	286	279	277	283	273.80	17.02
	Post-Treatmentment	282	286	322	312	290	298.40	17.57
	Baseline	301	321	310	291	322	309.00	13.25
	Post-Demineralizationeralization	253	249	259	258	257	255.20	4.15
	Post-Treatmentment	261	251	272	270	258	262.40	8.68

Treatment Group	Stage	HK1	HK2	HK3	HK4	HK5	Average	StnDev
	Baseline	276	281	279	286	278	280.00	3.81
	Post-Demineralization eralization	238	251	247	238	233	241.40	7.37
	Post-Treatmentment	261	243	267	236	253	252.00	12.69
	Baseline	312	281	296	295	299	296.60	11.06
	Post-Demineralization eralization	242	238	252	201	232	233.00	19.31
	Post-Treatmentment	247	237	234	266	241	245.00	12.71
	Baseline	340	345	313	333	300	326.20	19.04
	Post-Demineralization eralization	223	226	205	213	213	216.00	8.49
	Post-Treatmentment	261	292	267	278	291	277.80	13.92
	Baseline	258	258	264	254	249	256.60	5.55
	Post-Demineralization eralization	135	127	139	141	131	134.60	5.73
	Post-Treatmentment	140	149	162	158	157	153.20	8.76
**1% CaMgZnHAP**	Baseline	301	276	263	254	254	269.60	19.73
	Post-Demineralization eralization	161	151	159	169	148	157.60	8.35
	Post-Treatmentment	185	196	189	181	191	188.40	5.73
	Baseline	311	280	299	315	299	300.80	13.65
	Post-Demineralization eralization	244	237	241	231	212	233.00	12.71
	Post-Treatmentment	232	277	281	281	268	267.80	20.71
	Baseline	328	332	308	324	312	320.80	10.35
	Post-Demineralization	256	228	245	256	249	246.80	11.52

Treatment Group	Stage	HK1	HK2	HK3	HK4	HK5	Average	StnDev
	eralization							
	Post-Treatmentment	279	267	278	279	285	277.60	6.54
	Baseline	340	304	292	328	325	317.80	19.40
	Post-Demineralization eralization	280	241	259	249	246	255.00	15.44
	Post-Treatmentment	278	270	268	293	273	276.40	10.01
	Baseline	343	348	330	352	352	345.00	9.17
	Post-Demineralization eralization	249	270	277	279	253	265.60	13.81
	Post-Treatmentment	247	254	244	256	246	249.40	5.27
	Baseline	328	324	340	336	336	332.80	6.57
	Post-Demineralization eralization	235	233	241	219	216	228.80	10.78
	Post-Treatmentment	310	278	306	294	284	294.40	13.74
	Baseline	340	342	351	339	310	336.40	15.50
	Post-Demineralization eralization	248	256	267	251	258	256.00	7.31
	Post-Treatmentment	285	286	277	261	270	275.80	10.52
	Baseline	278	278	283	295	299	286.60	9.81
	Post-Demineralization eralization	240	239	226	205	211	224.20	15.93
	Post-Treatmentment	221	227	220	213	210	218.20	6.76
	Baseline	299	307	293	310	304	302.60	6.73
	Post-Demineralization eralization	215	217	215	233	225	221.00	7.87
	Post-Treatmentment	247	231	249	228	252	241.40	11.06

Treatment Group	Stage	HK1	HK2	HK3	HK4	HK5	Average	StnDev
	Baseline	374	404	381	388	374	384.20	12.50
	Post-Demineralization eralization	239	252	251	245	238	245.00	6.52
	Post-Treatmentment	291	294	272	286	288	286.20	8.50
**1% nanoXim**	Baseline	279	308	306	317	299	301.80	14.27
	Post-Demineralization eralization	191	199	203	204	216	202.60	9.07
	Post-Treatmentment	220	238	226	216	220	224.00	8.60
	Baseline	271	279	265	266	266	269.40	5.86
	Post-Demineralization eralization	229	242	228	221	213	226.60	10.74
	Post-Treatmentment	251	249	248	228	228	240.80	11.73
	Baseline	290	319	308	291	302	302.00	12.14
	Post-Demineralization eralization	201	243	229	236	232	228.20	16.08
	Post-Treatmentment	199	236	248	250	253	237.20	22.31
	Baseline	262	270	266	283	277	271.60	8.44
	Post-Demineralization eralization	169	199	196	209	210	196.60	16.59
	Post-Treatmentment	192	192	206	197	197	196.80	5.72
	Baseline	345	335	321	324	340	333.00	10.27
	Post-Demineralization eralization	205	212	206	191	202	203.20	7.73
	Post-Treatmentment	246	238	246	239	257	245.20	7.60
	Baseline	299	287	276	306	274	288.40	14.01
	Post-Demineralization	226	213	199	212	199	209.80	11.30

Treatment Group	Stage	HK1	HK2	HK3	HK4	HK5	Average	StnDev
	eralization							
	Post-Treatmentment	203	235	218	220	195	214.20	15.61
	Baseline	400	393	379	389	388	389.80	7.66
	Post-Demineralization eralization	254	236	249	258	264	252.20	10.59
	Post-Treatmentment	269	266	279	296	298	281.60	14.88
	Baseline	282	288	292	269	302	286.60	12.24
	Post-Demineralization eralization	216	219	219	203	209	213.20	7.01
	Post-Treatmentment	194	225	213	236	234	220.40	17.33
	Baseline	302	343	320	311	324	320.00	15.41
	Post-Demineralization eralization	249	220	244	229	244	237.20	12.19
	Post-Treatmentment	267	279	296	264	266	274.40	13.43
	Baseline	328	310	329	299	326	318.40	13.32
	Post-Demineralization eralization	256	269	223	264	266	255.60	18.85
	Post-Treatmentment	276	287	279	277	260	275.80	9.83
**1% Tricalcium Phosphate**	Baseline	282	316	295	298	277	293.60	15.27
	Post-Demineralization eralization	207	215	221	223	219	217.00	6.32
	Post-Treatmentment	232	220	228	255	245	236.00	13.95
	Baseline	354	349	377	364	345	357.80	12.87
	Post-Demineralization eralization	308	278	292	264	266	281.60	18.51
	Post-	285	255	292	278	295	281.00	15.95

Treatment Group	Stage	HK1	HK2	HK3	HK4	HK5	Average	StnDev
	Treatmentment							
	Baseline	330	328	333	355	304	330.00	18.12
	Post-Demineralization eralization	292	283	264	278	281	279.60	10.16
	Post-Treatmentment	287	308	315	292	290	298.40	12.34
	Baseline	326	340	345	351	364	345.20	13.99
	Post-Demineralization eralization	264	271	284	246	261	265.20	13.92
	Post-Treatmentment	264	278	266	274	288	274.00	9.70
	Baseline	263	294	296	280	265	279.60	15.53
	Post-Demineralization eralization	178	186	181	169	178	178.40	6.19
	Post-Treatmentment	175	188	186	186	181	183.20	5.26
	Baseline	296	321	283	291	296	297.40	14.22
	Post-Demineralization eralization	250	263	265	249	239	253.20	10.78
	Post-Treatmentment	267	265	248	242	221	248.60	18.80
	Baseline	256	259	245	259	278	259.40	11.89
	Post-Demineralization eralization	183	176	165	161	186	174.20	10.94
	Post-Treatmentment	191	186	191	187	189	188.80	2.28
	Baseline	320	326	304	316	301	313.40	10.62
	Post-Demineralization eralization	253	264	264	270	271	264.40	7.16
	Post-Treatmentment	278	269	277	279	285	277.60	5.73
	Baseline	322	333	325	328	311	323.80	8.23

Treatment Group	Stage	HK1	HK2	HK3	HK4	HK5	Average	StnDev
	Post-Demineralization eralization	250	257	258	254	256	255.00	3.16
	Post-Treatmentment	267	267	261	271	249	263.00	8.60
	Baseline	250	242	264	260	277	258.60	13.41
	Post-Demineralization eralization	188	203	192	181	171	187.00	11.98
	Post-Treatmentment	208	191	223	196	198	203.20	12.68
**Deionized water**	Baseline	312	343	326	285	315	316.20	21.25
	Post-Demineralization eralization	221	192	221	203	206	208.60	12.46
	Post-Treatmentment	197	211	207	197	216	205.60	8.47
	Baseline	320	300	290	280	292	296.40	14.99
	Post-Demineralization eralization	219	227	227	235	224	226.40	5.81
	Post-Treatmentment	211	232	218	225	228	222.80	8.35
	Baseline	295	328	295	290	273	296.20	19.94
	Post-Demineralization eralization	266	286	268	279	278	275.40	8.29
	Post-Treatmentment	298	274	284	273	291	284.00	10.79
	Baseline	283	285	291	278	270	281.40	7.89
	Post-Demineralization eralization	161	158	189	165	171	168.80	12.30
	Post-Treatmentment	181	165	176	167	164	170.60	7.50
	Baseline	352	361	335	351	348	349.40	9.40
	Post-Demineralization eralization	293	294	295	292	295	293.80	1.30

Treatment Group	Stage	HK1	HK2	HK3	HK4	HK5	Average	StnDev
	Post-Treatmentment	282	269	313	272	278	282.80	17.63
	Baseline	295	311	328	308	310	310.40	11.76
	Post-Demineralization eralization	242	242	242	245	247	243.60	2.30
	Post-Treatmentment	245	259	231	249	242	245.20	10.21
	Baseline	315	339	348	329	312	328.60	15.37
	Post-Demineralization eralization	254	256	249	254	255	253.60	2.70
	Post-Treatmentment	252	236	242	250	266	249.20	11.37
	Baseline	261	282	281	298	286	281.60	13.35
	Post-Demineralization eralization	225	221	211	205	217	215.80	7.95
	Post-Treatmentment	220	211	211	216	205	212.60	5.68
	Baseline	352	340	357	354	337	348.00	8.92
	Post-Demineralization eralization	287	287	296	278	281	285.80	6.91
	Post-Treatmentment	287	272	273	260	271	272.60	9.61
	Baseline	287	277	307	272	268	282.20	15.58
	Post-Demineralization eralization	194	196	194	208	206	199.60	6.84
	Post-Treatmentment	188	195	199	199	199	196.00	4.80

HK- Hardness Knoop

Table S2. Hardness Knoop average values of each enamel sample (first experiment, assessment of remineralizing efficacy of various calcium sources).

.

Treatment Group	Average Baseline HK	Average Post-Demineralizationeralization HK	Average Post-Treatmentmentment HK
**1450 ppm F**	347.75	304.20	341.80
	311.00	202.80	228.20
	256.60	198.80	227.80
	350.00	227.80	252.20
	329.00	273.80	298.40
	309.00	255.20	262.40
	280.00	241.40	252.00
	296.60	233.00	245.00
	326.20	216.00	277.80
	256.60	134.60	153.20
**1% CaMgZnHAP**	269.60	157.60	188.40
	300.80	233.00	267.80
	320.80	246.80	277.60
	317.80	255.00	276.40
	345.00	265.60	249.40
	332.80	228.80	294.40
	336.40	256.00	275.80
	286.60	224.20	218.20
	302.60	221.00	241.40
	384.20	245.00	286.20
**1% nanoXim**	301.80	202.60	224.00
	269.40	226.60	240.80
	302.00	228.20	237.20
	271.60	196.60	196.80
	333.00	203.20	245.20
	288.40	209.80	214.20
	389.80	252.20	281.60
	286.60	213.20	220.40

Treatment Group	Average Baseline HK	Average Post-Demineralizationeralization HK	Average Post-Treatmentmentment HK
	320.00	237.20	274.40
	318.40	255.60	275.80
**1% Tricalcium Phosphate**	293.60	217.00	236.00
	357.80	281.60	281.00
	330.00	279.60	298.40
	345.20	265.20	274.00
	279.60	178.40	183.20
	297.40	253.20	248.60
	259.40	174.20	188.80
	313.40	264.40	277.60
	323.80	255.00	263.00
	258.60	187.00	203.20
**Deionized water**	316.20	208.60	205.60
	296.40	226.40	222.80
	296.20	275.40	284.00
	281.40	168.80	170.60
	349.40	293.80	282.80
	310.40	243.60	245.20
	328.60	253.60	249.20
	281.60	215.80	212.60
	348.00	285.80	272.60
	282.20	199.60	196.00

Table S3. Percentage of surface microhardness recovery (%SMHR) calculated for each enamel sample, average %SMHR and standard deviations StnDev calculated for treatment groups (first experiment, assessment of remineralizing efficacy of various calcium sources).

Treatment group	% SMHR	Average % SMHR	StnDev (% SMHR)
**1450 ppm F**	86.34	**35.56**	**23.41**

Treatment group	% SMHR	Average % SMHR	StnDev (% SMHR)
	23.48		
	50.17		
	19.97		
	44.57		
	13.38		
	27.46		
	18.87		
	56.08		
	15.25		
**1% CaMgZnHAP**	27.50	**26.68**	**25.30**
	51.33		
	41.62		
	34.08		
	-20.40		
	63.08		
	24.63		
	-9.62		
	25.00		
	29.60		
**1% nanoXim**	21.57	**21.34**	**14.31**
	33.18		
	12.20		
	0.27		
	32.36		
	5.60		
	21.37		
	9.81		
	44.93		
	32.17		

Treatment group	% SMHR	Average % SMHR	StnDev (% SMHR)
**1% Tricalcium Phosphate**	24.80	**14.50**	**14.23**
	-0.79		
	37.30		
	11.00		
	4.74		
	-10.41		
	17.14		
	26.94		
	11.63		
	22.63		
**Deionized water**	-2.79	**-1.87**	**17.11**
	-5.14		
	41.35		
	1.60		
	-19.78		
	2.40		
	-5.87		
	-4.86		
	-21.22		
	-4.36		

Table S4. Second experiment surface microhardness raw data (assessment of remineralizing efficacy of aspartic acid at varying concentrations and in combination with tricalcium phosphate).

Treatment Group	Stage	HK1	HK2	HK3	HK4	HK5	Average	StnDev
**Aspartic Acid 0.5%**	Baseline	265	272	273	274	266	270.00	4.18
	Post-Demineralization	153	162	153	153	155	155.20	3.90
	Post-Treatment	128	132	133	131	137	132.20	3.27

Treatment Group	Stage	HK1	HK2	HK3	HK4	HK5	Average	StnDev
	Baseline	300	304	308	304	301	303.40	3.13
	Post-Demineralization	136	134	133	138	136	135.40	1.95
	Post-Treatment	71	78	80	82	75	77.20	4.32
	Baseline	304	302	304	304	306	304.00	1.41
	Post-Demineralization	207	205	215	207	205	207.80	4.15
	Post-Treatment	131	146	129	144	137	137.40	7.57
	Baseline	326	326	322	320	330	324.80	3.90
	Post-Demineralization	204	210	207	207	205	206.60	2.30
	Post-Treatment	130	146	142	134	135	137.40	6.47
	Baseline	336	340	343	340	343	340.40	2.88
	Post-Demineralization	199	203	202	201	207	202.40	2.97
	Post-Treatment	167	165	163	156	152	160.60	6.35
	Baseline	339	342	343	340	342	341.20	1.64
	Post-Demineralization	144	153	166	164	161	157.60	9.07
	Post-Treatment	166	179	161	172	179	171.40	7.96
	Baseline	355	360	361	360	360	359.20	2.39
	Post-Demineralization	256	269	261	259	257	260.40	5.18
	Post-Treatment	186	193	206	210	195	198.00	9.82
	Baseline	360	357	361	355	363	359.20	3.19
	Post-Demineralization	227	223	226	218	222	223.20	3.56
	Post-Treatment	181	173	175	175	172	175.20	3.49
	Baseline	381	388	377	377	381	380.80	4.49
	Post-Demineralization	192	213	213	213	192	204.60	11.50
	Post-Treatment	145	157	156	151	165	154.80	7.43
**Aspartic Acid**	Baseline	270	279	278	272	277	275.20	3.96

Treatment Group	Stage	HK1	HK2	HK3	HK4	HK5	Average	StnDev
**0.5% + Tricalcium Phosphate 1.0%**	Post-Demineralization	172	167	174	171	171	171.00	2.55
	Post-Treatment	197	209	195	199	211	202.20	7.29
	Baseline	301	307	301	301	306	303.20	3.03
	Post-Demineralization	164	156	150	164	166	160.00	6.78
	Post-Treatment	201	189	194	195	193	194.40	4.34
	Baseline	306	300	305	305	306	304.40	2.51
	Post-Demineralization	185	189	194	191	185	188.80	3.90
	Post-Treatment	212	226	220	225	221	220.80	5.54
	Baseline	300	329	333	332	329	324.60	13.87
	Post-Demineralization	225	237	236	224	236	231.60	6.50
	Post-Treatment	250	250	246	261	260	253.40	6.69
	Baseline	319	326	329	328	329	326.20	4.21
	Post-Demineralization	161	175	173	179	178	173.20	7.22
	Post-Treatment	195	210	209	207	214	207.00	7.18
	Baseline	340	337	343	336	340	339.20	2.77
	Post-Demineralization	246	253	258	249	255	252.20	4.76
	Post-Treatment	306	298	302	296	305	301.40	4.34
	Baseline	335	345	343	342	345	342.00	4.12
	Post-Demineralization	276	272	280	286	286	280.00	6.16
	Post-Treatment	328	328	325	326	326	326.60	1.34
	Baseline	357	355	363	360	361	359.20	3.19
	Post-Demineralization	270	281	276	287	272	277.20	6.91
	Post-Treatment	325	313	319	322	315	318.80	4.92
	Baseline	364	361	360	361	360	361.20	1.64

Treatment Group	Stage	HK1	HK2	HK3	HK4	HK5	Average	StnDev
	Post-Demineralization	215	224	226	224	225	222.80	4.44
	Post-Treatment	319	311	315	312	317	314.80	3.35
	Baseline	379	381	379	374	381	378.80	2.86
	Post-Demineralization	206	209	200	205	200	204.00	3.94
	Post-Treatment	280	276	283	286	281	281.20	3.70
**Aspartic Acid 0.1% + Tricalcium Phosphate 0.5%**	Baseline	287	282	281	287	286	284.60	2.88
	Post-Demineralization	163	164	149	168	173	163.40	8.96
	Post-Treatment	206	202	204	205	204	204.20	1.48
	Baseline	301	300	299	298	302	300.00	1.58
	Post-Demineralization	205	209	211	232	224	216.20	11.34
	Post-Treatment	271	264	264	262	262	264.60	3.71
	Baseline	315	312	310	308	315	312.00	3.08
	Post-Demineralization	184	166	178	173	173	174.80	6.69
	Post-Treatment	269	269	266	271	267	268.40	1.95
	Baseline	317	315	315	322	321	318.00	3.32
	Post-Demineralization	220	228	223	221	224	223.20	3.11
	Post-Treatment	225	235	243	226	233	232.40	7.33
	Baseline	328	332	333	329	330	330.40	2.07
	Post-Demineralization	149	132	140	136	146	140.60	6.99
	Post-Treatment	175	179	186	182	181	180.60	4.04
	Baseline	332	335	342	333	339	336.20	4.21
	Post-Demineralization	174	168	175	185	184	177.20	7.19
	Post-Treatment	197	213	209	228	201	209.60	12.07
	Baseline	346	340	345	348	346	345.00	3.00
	Post-	150	150	173	162	153	157.60	9.91

Treatment Group	Stage	HK1	HK2	HK3	HK4	HK5	Average	StnDev
	Demineralization							
	Post-Treatment	217	221	205	216	215	214.80	5.93
	Baseline	349	351	352	348	352	350.40	1.82
	Post-Demineralization	175	181	181	176	175	177.60	3.13
	Post-Treatment	216	227	234	221	219	223.40	7.16
	Baseline	369	372	366	364	360	366.20	4.60
	Post-Demineralization	160	170	151	174	161	163.20	9.04
	Post-Treatment	231	245	247	252	252	245.40	8.62
	Baseline	374	377	371	369	374	373.00	3.08
	Post-Demineralization	201	196	208	219	222	209.20	11.21
	Post-Treatment	227	232	232	225	236	230.40	4.39
**Aspartic Acid 0.1%**	Baseline	282	281	288	284	282	283.40	2.79
	Post-Demineralization	124	116	127	115	117	119.80	5.36
	Post-Treatment	119	122	117	121	121	120.00	2.00
	Baseline	296	299	301	301	304	300.20	2.95
	Post-Demineralization	195	171	161	185	185	179.40	13.37
	Post-Treatment	195	200	201	184	182	192.40	8.91
	Baseline	306	312	310	304	311	308.60	3.44
	Post-Demineralization	182	183	185	190	194	186.80	5.07
	Post-Treatment	205	205	204	196	205	203.00	3.94
	Baseline	319	324	325	317	325	322.00	3.74
	Post-Demineralization	162	170	161	164	164	164.20	3.49
	Post-Treatment	188	208	201	194	200	198.20	7.56
	Baseline	326	333	330	330	328	329.40	2.61
	Post-	151	156	155	148	153	152.60	3.21

Treatment Group	Stage	HK1	HK2	HK3	HK4	HK5	Average	StnDev
	Demineralization							
	Post-Treatment	158	157	152	152	153	154.40	2.88
	Baseline	339	339	335	332	339	336.80	3.19
	Post-Demineralization	186	180	195	182	191	186.80	6.22
	Post-Treatment	189	183	179	175	191	183.40	6.69
	Baseline	339	345	346	346	346	344.40	3.05
	Post-Demineralization	229	212	231	235	222	225.80	9.04
	Post-Treatment	216	207	220	211	210	212.80	5.17
	Baseline	351	349	352	352	349	350.60	1.52
	Post-Demineralization	162	147	147	166	157	155.80	8.64
	Post-Treatment	145	145	162	156	145	150.60	7.96
	Baseline	368	368	364	366	364	366.00	2.00
	Post-Demineralization	209	218	211	216	225	215.80	6.30
	Post-Treatment	225	221	213	212	219	218.00	5.48
	Baseline	376	372	376	371	371	373.20	2.59
	Post-Demineralization	156	168	170	179	162	167.00	8.66
	Post-Treatment	167	168	161	170	165	166.20	3.42
**1450 ppm Fluoride**	Baseline	274	278	274	277	277	276.00	1.87
	Post-Demineralization	102	120	120	105	106	110.60	8.71
	Post-Treatment	156	151	157	138	146	149.60	7.83
	Baseline	302	300	302	304	304	302.40	1.67
	Post-Demineralization	155	158	146	159	180	159.60	12.50
	Post-Treatment	168	179	175	182	174	175.60	5.32
	Baseline	305	300	304	310	304	304.60	3.58
	Post-Demineralization	137	124	135	154	145	139.00	11.25

Treatment Group	Stage	HK1	HK2	HK3	HK4	HK5	Average	StnDev
	Post-Treatment	170	182	180	174	179	177.00	4.90
	Baseline	329	325	324	324	321	324.60	2.88
	Post-Demineralization	219	220	218	224	220	220.20	2.28
	Post-Treatment	238	244	235	232	235	236.80	4.55
	Baseline	325	322	332	330	324	326.60	4.22
	Post-Demineralization	184	197	185	197	190	190.60	6.27
	Post-Treatment	199	188	196	198	200	196.20	4.82
	Baseline	339	333	342	337	340	338.20	3.42
	Post-Demineralization	213	229	231	228	238	227.80	9.15
	Post-Treatment	280	278	272	270	283	276.60	5.46
	Baseline	345	337	343	345	342	342.40	3.29
	Post-Demineralization	161	169	163	168	156	163.40	5.32
	Post-Treatment	184	177	172	172	184	177.80	6.02
	Baseline	360	354	360	358	358	358.00	2.45
	Post-Demineralization	178	180	196	194	196	188.80	9.01
	Post-Treatment	201	201	202	200	203	201.40	1.14
	Baseline	361	368	366	363	364	364.40	2.70
	Post-Demineralization	198	195	176	178	181	185.60	10.16
	Post-Treatment	186	184	172	186	172	180.00	7.35
	Baseline	379	379	372	376	374	376.00	3.08
	Post-Demineralization	207	199	182	188	199	195.00	9.92
	Post-Treatment	205	213	209	204	206	207.40	3.65
**Deionised Water**	Baseline	284	280	278	278	279	279.80	2.49
	Post-Demineralization	136	145	152	145	143	144.20	5.72
	Post-Treatment	118	111	111	124	121	117.00	5.87

Treatment Group	Stage	HK1	HK2	HK3	HK4	HK5	Average	StnDev
	Baseline	304	300	300	302	301	301.40	1.67
	Post-Demineralization	144	148	151	139	140	144.40	5.13
	Post-Treatment	136	140	134	140	133	136.60	3.29
	Baseline	305	308	304	302	304	304.60	2.19
	Post-Demineralization	152	156	157	167	152	156.80	6.14
	Post-Treatment	149	156	153	148	153	151.80	3.27
	Baseline	324	326	322	325	325	324.40	1.52
	Post-Demineralization	124	139	135	137	145	136.00	7.68
	Post-Treatment	150	146	142	152	153	148.60	4.56
	Baseline	326	330	329	329	326	328.00	1.87
	Post-Demineralization	199	186	209	196	207	199.40	9.24
	Post-Treatment	174	172	167	174	176	172.60	3.44
	Baseline	339	336	333	339	340	337.40	2.88
	Post-Demineralization	180	194	195	185	188	188.40	6.27
	Post-Treatment	148	163	161	149	151	154.40	7.06
	Baseline	342	345	345	340	343	343.00	2.12
	Post-Demineralization	168	161	181	182	177	173.80	9.04
	Post-Treatment	156	169	152	150	160	157.40	7.54
	Baseline	355	361	358	355	358	357.40	2.51
	Post-Demineralization	170	175	169	180	189	176.60	8.20
	Post-Treatment	150	143	154	154	157	151.60	5.41
	Baseline	369	366	363	364	364	365.20	2.39
	Post-Demineralization	268	261	265	269	261	264.80	3.77
	Post-Treatment	189	172	188	191	186	185.20	7.60
	Baseline	376	377	374	377	374	375.60	1.52

Treatment Group	Stage	HK1	HK2	HK3	HK4	HK5	Average	StnDev
	Post-Demineralization	226	225	225	232	231	227.80	3.42
	Post-Treatment	213	222	211	222	219	217.40	5.13

Table S5. Hardness Knoop average values of each enamel sample (second experiment, assessment of remineralizing efficacy of aspartic acid at varying concentrations and in combination with tricalcium phosphate).

Treatment Group	Average Baseline HK	Average Post-Demineralization HK	Average Post-Treatment HK
**Aspartic Acid 0.5%**	270.00	155.20	132.20
	303.40	135.40	77.20
	304.00	207.80	137.40
	331.44	204.60	154.80
	324.80	206.60	137.40
	340.40	202.40	160.60
	341.20	157.60	171.40
	359.20	260.40	198.00
	359.20	223.20	175.20
	380.80	204.60	154.80
**Aspartic Acid 0.5% + Tricalcium Phosphate 1.0%**	275.20	171.00	202.20
	303.20	160.00	194.40
	304.40	188.80	220.80
	324.60	231.60	253.40
	326.20	173.20	207.00
	339.20	252.20	301.40
	342.00	280.00	326.60
	359.20	277.20	318.80
	361.20	222.80	314.80
	378.80	204.00	281.20
**Aspartic Acid 0.1% +**	284.60	163.40	204.20

Treatment Group	Average Baseline HK	Average Post-Demineralization HK	Average Post-Treatment HK
**Tricalcium Phosphate 0.5%**	300.00	216.20	264.60
	312.00	174.80	268.40
	318.00	223.20	232.40
	330.40	140.60	180.60
	336.20	177.20	209.60
	345.00	157.60	214.80
	350.40	177.60	223.40
	366.20	163.20	245.40
	373.00	209.20	230.40
**1450 ppm Fluoride**	276.00	110.60	149.60
	302.40	159.60	175.60
	304.60	139.00	177.00
	324.60	220.20	236.80
	326.60	190.60	196.20
	338.20	227.80	276.60
	342.40	163.40	177.80
	358.00	188.80	201.40
	364.40	185.60	180.00
	376.00	195.00	207.40
**Aspartic Acid 0.1%**	283.40	119.80	120.00
	300.20	179.40	192.40
	308.60	186.80	203.00
	322.00	164.20	198.20
	329.40	152.60	154.40
	336.80	186.80	183.40
	344.40	225.80	212.80
	350.60	155.80	150.60
	366.00	215.80	218.00

Treatment Group	Average Baseline HK	Average Post-Demineralization HK	Average Post-Treatment HK
	373.20	167.00	166.20
**Deionised Water**	279.80	144.20	117.00
	301.40	144.40	136.60
	304.60	156.80	151.80
	324.40	136.00	148.60
	328.00	199.40	172.60
	337.40	188.40	154.40
	343.00	173.80	157.40
	357.40	176.60	151.60
	365.20	264.80	185.20
	375.60	227.80	217.40

Table S6. Percentage of surface microhardness recovery (%SMHR) calculated for each enamel sample, average %SMHR and standard deviations (StnDev) calculated for treatment groups (second experiment, assessment of remineralizing efficacy of aspartic acid at varying concentrations and in combination with tricalcium phosphate).

Treatment Group	% SMHR	Average % SMHR	StnDev (% SMHR)
**Aspartic Acid 0.5%**	-20.03	**-37.32**	24.64
	-34.64		
	-73.18		
	-37.32		
	-58.54		
	-30.29		
	7.52		
	-63.16		
	-35.29		
	-28.26		
**Aspartic Acid 0.5% + Tricalcium Phosphate 1.0%**	29.94	**42.03**	19.45
	24.02		

Treatment Group	% SMHR	Average % SMHR	StnDev (% SMHR)
	27.68		
	23.44		
	22.09		
	56.55		
	75.16		
	50.73		
	66.47		
	44.16		
**Aspartic Acid 0.1% + Tricalcium Phosphate 0.5%**	33.66	**32.13**	18.85
	57.76		
	68.22		
	9.70		
	21.07		
	20.38		
	30.52		
	26.50		
	40.49		
	12.94		
**1450 ppm Fluoride**	23.58	**14.12**	13.40
	11.20		
	22.95		
	15.90		
	4.12		
	44.20		
	8.04		
	7.45		
	-3.13		
	6.85		
**Aspartic Acid 0.1%**	0.12	**3.19**	9.37

Treatment Group	% SMHR	Average % SMHR	StnDev (% SMHR)
	10.76		
	13.30		
	21.55		
	1.02		
	-2.27		
	-10.96		
	-2.67		
	1.46		
	-0.39		
**Deionised Water**	-20.06	**-17.52**	23.54
	-4.97		
	-3.38		
	6.69		
	-20.84		
	-22.82		
	-9.69		
	-13.83		
	-79.28		
	-7.04		

Table S7. Third experiment surface microhardness raw data (assessment of remineralizing potential of aspartic acid combined with diverse calcium sources).

Treatment Group	Stage	HK1	HK2	HK3	HK4	HK5	Average	StnDev
0.5% Aspartic acid + 1% Dicalcium phosphate	Baseline	307	292	316	330	340	317.00	18.87
	Post-Demineralization	163	158	185	167	169	168.40	10.19
	Post-Treatment	253	249	243	255	242	248.40	5.81
	Baseline	313	330	312	324	321	320.00	7.58
	Post-Demineralization	147	135	153	140	166	148.20	12.07

Treatment Group	Stage	HK1	HK2	HK3	HK4	HK5	Average	StnDev
	Post-Treatment	333	311	321	335	321	324.20	9.86
	Baseline	361	324	332	335	326	335.60	14.88
	Post-Demineralization	163	166	161	178	166	166.80	6.61
	Post-Treatment	232	238	225	231	221	229.40	6.58
	Baseline	313	357	349	326	357	340.40	19.89
	Post-Demineralization	167	155	178	169	183	170.40	10.81
	Post-Treatment	293	277	274	307	284	287.00	13.36
	Baseline	369	355	340	358	368	358.00	11.77
	Post-Demineralization	294	284	280	276	291	285.00	7.48
	Post-Treatment	321	316	329	299	305	314.00	12.08
	Baseline	368	357	363	371	360	363.80	5.72
	Post-Demineralization	183	172	175	201	189	184.00	11.62
	Post-Treatment	225	222	219	205	211	216.40	8.23
	Baseline	364	358	369	389	376	371.20	11.95
	Post-Demineralization	168	186	173	151	150	165.60	15.27
	Post-Treatment	209	210	221	213	197	210.00	8.66
	Baseline	374	360	379	384	372	373.80	9.01
	Post-Demineralization	174	180	190	195	170	181.80	10.55
	Post-Treatment	267	260	250	242	252	254.20	9.60
	Baseline	398	374	371	395	388	385.20	12.19
	Post-Demineralization	209	209	216	219	209	212.40	4.77
	Post-Treatment	267	280	276	265	285	274.60	8.50
	Baseline	391	396	381	398	395	392.20	6.76
	Post-Demineralization	236	242	245	235	263	244.20	11.30
	Post-Treatment	315	313	291	287	306	302.40	12.76

Treatment Group	Stage	HK1	HK2	HK3	HK4	HK5	Average	StnDev
0.5% Aspartic acid + 1% CaMgZn	Baseline	306	310	325	313	300	310.80	9.31
	Post-Demineralization	128	126	124	127	141	129.20	6.76
	Post-Treatment	175	161	154	159	163	162.40	7.80
	Baseline	300	330	333	333	325	324.20	13.92
	Post-Demineralization	210	213	212	179	177	198.20	18.49
	Post-Treatment	226	225	226	209	218	220.80	7.40
	Baseline	388	374	328	345	358	358.60	23.58
	Post-Demineralization	232	216	225	229	232	226.80	6.69
	Post-Treatment	245	240	247	242	254	245.60	5.41
	Baseline	357	360	339	342	351	349.80	9.15
	Post-Demineralization	221	229	227	242	249	233.60	11.52
	Post-Treatment	269	262	274	292	263	272.00	12.19
	Baseline	360	357	349	352	355	354.60	4.28
	Post-Demineralization	194	221	221	247	229	222.40	19.10
	Post-Treatment	260	268	264	265	296	270.60	14.48
	Baseline	345	381	364	382	360	366.40	15.50
	Post-Demineralization	284	295	304	249	282	282.80	20.87
	Post-Treatment	328	328	364	324	329	334.60	16.55
	Baseline	355	395	386	346	360	368.40	21.03
	Post-Demineralization	211	185	195	228	177	199.20	20.50
	Post-Treatment	291	253	301	271	282	279.60	18.54
	Baseline	382	374	389	371	377	378.60	7.09
	Post-Demineralization	219	238	237	261	243	239.60	15.03
	Post-Treatment	294	304	294	300	298	298.00	4.24
	Baseline	386	384	381	382	374	381.40	4.56

Treatment Group	Stage	HK1	HK2	HK3	HK4	HK5	Average	StnDev
	Post-Demineralization	250	232	270	291	291	266.80	25.86
	Post-Treatment	292	310	305	282	296	297.00	11.00
	Baseline	402	409	396	391	409	401.40	7.96
	Post-Demineralization	248	234	250	235	241	241.60	7.30
	Post-Treatment	291	298	284	299	299	294.20	6.61
0.5% Aspartic acid + 1% nanoXim®- CarePaste Hydroxyapatite (nano)	Baseline	325	315	301	313	302	311.20	9.96
	Post-Demineralization	222	205	216	200	225	213.60	10.78
	Post-Treatment	271	263	261	268	284	269.40	9.07
	Baseline	317	343	328	312	310	322.00	13.66
	Post-Demineralization	173	172	167	174	167	170.60	3.36
	Post-Treatment	192	212	216	208	199	205.40	9.79
	Baseline	349	307	332	335	342	333.00	15.95
	Post-Demineralization	172	175	153	160	176	167.20	10.18
	Post-Treatment	201	205	196	211	188	200.20	8.76
	Baseline	358	340	332	360	328	343.60	14.72
	Post-Demineralization	147	171	165	189	172	168.80	15.11
	Post-Treatment	220	257	239	236	232	236.80	13.41
	Baseline	369	330	357	372	351	355.80	16.78
	Post-Demineralization	163	175	175	170	156	167.80	8.23
	Post-Treatment	217	223	225	216	208	217.80	6.69
	Baseline	364	368	371	371	351	365.00	8.34
	Post-Demineralization	254	261	244	251	259	253.80	6.76
	Post-Treatment	265	285	273	282	256	272.20	11.99
	Baseline	376	366	366	361	377	369.20	6.98
	Post-	126	114	123	122	141	125.20	9.88

Treatment Group	Stage	HK1	HK2	HK3	HK4	HK5	Average	StnDev
	Demineralization							
	Post-Treatment	192	170	166	183	200	182.20	14.36
	Baseline	393	391	400	402	382	393.60	7.96
	Post-Demineralization	261	221	234	259	260	247.00	18.40
	Post-Treatment	268	247	258	253	244	254.00	9.51
	Baseline	391	371	376	396	381	383.00	10.37
	Post-Demineralization	284	281	248	279	267	271.80	14.79
	Post-Treatment	293	299	299	299	300	298.00	2.83
	Baseline	406	415	374	388	396	395.80	15.88
	Post-Demineralization	261	268	240	258	247	254.80	11.21
	Post-Treatment	276	290	286	302	316	294.00	15.43
1% Aspartic acid + 1.5% Tricalcium Phosphate (TCP)	Baseline	320	310	308	310	316	312.80	5.02
	Post-Demineralization	177	162	155	179	168	168.20	10.08
	Post-Treatment	173	209	215	195	197	197.80	16.16
	Baseline	336	319	329	299	325	321.60	14.06
	Post-Demineralization	175	161	150	142	142	154.00	14.09
	Post-Treatment	198	175	172	175	184	180.80	10.62
	Baseline	326	325	342	333	345	334.20	9.09
	Post-Demineralization	188	169	173	214	185	185.80	17.66
	Post-Treatment	249	223	219	210	200	220.20	18.38
	Baseline	364	333	343	339	336	343.00	12.31
	Post-Demineralization	201	197	225	210	229	212.40	14.21
	Post-Treatment	258	264	250	269	244	257.00	10.15
	Baseline	368	354	349	360	352	356.60	7.54
	Post-Demineralization	219	220	223	232	221	223.00	5.24

Treatment Group	Stage	HK1	HK2	HK3	HK4	HK5	Average	StnDev
	Post-Treatment	267	263	261	232	253	255.20	13.94
	Baseline	337	361	381	366	377	364.40	17.31
	Post-Demineralization	186	207	188	166	163	182.00	17.99
	Post-Treatment	201	216	207	232	257	222.60	22.50
	Baseline	349	357	376	386	384	370.40	16.56
	Post-Demineralization	184	172	180	209	180	185.00	14.11
	Post-Treatment	264	266	253	254	222	251.80	17.64
	Baseline	364	388	381	369	371	374.60	9.71
	Post-Demineralization	205	235	219	194	220	214.60	15.66
	Post-Treatment	230	239	224	227	225	229.00	6.04
	Baseline	388	382	398	377	371	383.20	10.38
	Post-Demineralization	174	199	228	210	184	199.00	21.28
	Post-Treatment	249	243	250	248	259	249.80	5.81
	Baseline	402	402	386	395	393	395.60	6.73
	Post-Demineralization	207	225	225	205	213	215.00	9.59
	Post-Treatment	236	227	230	240	256	237.80	11.37
0,2% Aspartic Acid in artificial saliva	Baseline	328	355	332	340	315	334.00	14.92
	Post-Demineralization	172	165	194	188	158	175.40	15.01
	Post-Treatment	205	198	212	195	206	203.20	7.05
	Baseline	350	322	328	345	335	336.00	11.42
	Post-Demineralization	170	185	192	164	178	177.80	11.37
	Post-Treatment	215	208	225	202	217	213.40	9.07
	Baseline	334	358	322	345	362	344.20	16.48
	Post-Demineralization	160	175	182	155	168	168.00	11.07

Treatment Group	Stage	HK1	HK2	HK3	HK4	HK5	Average	StnDev
	Post-Treatment	178	175	182	169	180	176.80	5.22
	Baseline	355	342	368	335	360	352.00	13.13
	Post-Demineralization	182	175	194	168	188	181.40	10.33
	Post-Treatment	205	198	212	195	208	203.60	7.30
	Baseline	360	348	335	352	365	352.00	11.58
	Post-Demineralization	208	195	215	188	202	201.60	10.74
	Post-Treatment	245	238	252	240	248	244.60	5.81
	Baseline	370	338	345	363	350	353.20	12.87
	Post-Demineralization	224	222	196	175	185	200.40	21.84
	Post-Treatment	215	208	222	205	217	213.40	6.99
	Baseline	375	342	358	366	335	355.20	16.33
	Post-Demineralization	205	238	182	214	195	206.80	21.28
	Post-Treatment	238	232	245	225	239	235.80	7.60
	Baseline	390	382	358	365	374	373.80	12.79
	Post-Demineralization	220	215	198	205	212	210.00	8.80
	Post-Treatment	222	218	225	212	226	220.60	5.59
	Baseline	374	365	388	392	358	375.40	14.36
	Post-Demineralization	212	225	230	218	205	218.00	9.92
	Post-Treatment	258	265	252	260	256	258.20	4.97
	Baseline	382	395	368	374	388	381.40	10.85
	Post-Demineralization	235	222	244	215	228	228.80	11.26
	Post-Treatment	255	248	252	260	247	252.40	5.37
1450 ppm F (positive control)	Baseline	315	308	339	325	300	317.40	15.18
	Post-Demineralization	215	223	222	226	208	218.80	7.26

Treatment Group	Stage	HK1	HK2	HK3	HK4	HK5	Average	StnDev
	Post-Treatment	232	218	214	228	230	224.40	7.92
	Baseline	298	310	319	337	333	319.40	16.13
	Post-Demineralization	201	178	172	180	174	181.00	11.62
	Post-Treatment	173	181	191	188	171	180.80	8.84
	Baseline	342	340	315	337	348	336.40	12.62
	Post-Demineralization	220	203	211	216	207	211.40	6.80
	Post-Treatment	205	204	215	215	219	211.60	6.69
	Baseline	346	355	321	328	335	337.00	13.66
	Post-Demineralization	223	233	222	232	240	230.00	7.52
	Post-Treatment	229	231	236	247	251	238.80	9.76
	Baseline	369	352	381	363	337	360.40	16.76
	Post-Demineralization	186	186	174	189	195	186.00	7.65
	Post-Treatment	189	196	199	205	180	193.80	9.63
	Baseline	363	377	351	351	368	362.00	11.22
	Post-Demineralization	211	231	242	243	246	234.60	14.36
	Post-Treatment	238	238	234	236	236	236.40	1.67
	Baseline	346	372	388	382	369	371.40	16.12
	Post-Demineralization	180	197	203	186	209	195.00	11.94
	Post-Treatment	216	226	215	221	203	216.20	8.58
	Baseline	374	379	346	374	393	373.20	17.08
	Post-Demineralization	193	220	202	176	182	194.60	17.37
	Post-Treatment	207	186	207	216	207	204.60	11.10
	Baseline	374	395	421	371	366	385.40	22.77
	Post-Demineralization	229	204	195	182	185	199.00	18.88
	Post-Treatment	216	207	210	194	175	200.40	16.32

Treatment Group	Stage	HK1	HK2	HK3	HK4	HK5	Average	StnDev
	Baseline	400	391	376	381	398	389.20	10.47
	Post-Demineralization	214	211	210	227	236	219.60	11.41
	Post-Treatment	247	238	258	235	233	242.20	10.33
0.5% Aspartic acid + Monofluorophosphate 1450 ppm	Baseline	296	290	313	333	332	312.80	19.87
	Post-Demineralization	195	179	160	169	165	173.60	13.85
	Post-Treatment	171	173	171	173	197	177.00	11.22
	Baseline	300	325	325	328	329	321.40	12.10
	Post-Demineralization	194	160	159	139	139	158.20	22.49
	Post-Treatment	144	156	160	170	178	161.60	13.07
	Baseline	368	343	329	310	324	334.80	21.99
	Post-Demineralization	173	150	144	137	170	154.80	15.96
	Post-Treatment	143	136	143	121	149	138.40	10.76
	Baseline	326	358	330	358	342	342.80	15.07
	Post-Demineralization	228	222	239	228	226	228.60	6.31
	Post-Treatment	212	216	221	213	203	213.00	6.60
	Baseline	360	342	343	384	355	356.80	17.05
	Post-Demineralization	189	184	175	185	161	178.80	11.19
	Post-Treatment	159	157	140	141	165	152.40	11.26
	Baseline	340	376	371	360	374	364.20	14.87
	Post-Demineralization	170	172	186	186	189	180.60	8.88
	Post-Treatment	156	162	168	150	174	162.00	9.49
	Baseline	371	396	376	352	357	370.40	17.36
	Post-Demineralization	107	122	111	124	119	116.60	7.30
	Post-Treatment	121	128	132	110	128	123.80	8.67
	Baseline	379	371	381	363	377	374.20	7.29

Treatment Group	Stage	HK1	HK2	HK3	HK4	HK5	Average	StnDev
	Post-Demineralization	182	195	198	207	194	195.20	8.98
	Post-Treatment	163	147	167	153	166	159.20	8.79
	Baseline	388	374	376	398	382	383.60	9.74
	Post-Demineralization	247	278	261	242	236	252.80	16.84
	Post-Treatment	219	230	208	203	201	212.20	12.15
	Baseline	417	421	368	409	382	399.40	23.22
	Post-Demineralization	232	216	214	204	213	215.80	10.16
	Post-Treatment	183	183	176	181	180	180.60	2.88
Deionized water (negative control)	Baseline	325	316	320	298	333	318.40	13.05
	Post-Demineralization	206	186	172	218	175	191.40	19.97
	Post-Treatment	135	137	146	163	135	143.20	11.97
	Baseline	316	312	322	336	311	319.40	10.24
	Post-Demineralization	191	201	184	188	203	193.40	8.26
	Post-Treatment	158	145	148	160	145	151.20	7.26
	Baseline	333	324	340	349	337	336.60	9.18
	Post-Demineralization	178	151	158	154	178	163.80	13.20
	Post-Treatment	133	127	147	140	140	137.40	7.64
	Baseline	358	320	335	313	358	336.80	20.92
	Post-Demineralization	160	184	160	181	160	169.00	12.37
	Post-Treatment	109	123	130	130	120	122.40	8.68
	Baseline	336	351	369	386	361	360.60	18.80
	Post-Demineralization	138	128	166	122	138	138.40	16.88
	Post-Treatment	112	133	104	122	113	116.80	11.08
	Baseline	377	376	372	351	332	361.60	19.63
	Post-	172	173	184	166	167	172.40	7.16

Treatment Group	Stage	HK1	HK2	HK3	HK4	HK5	Average	StnDev
	Demineralization							
	Post-Treatment	127	132	129	120	114	124.40	7.30
	Baseline	382	343	371	377	384	371.40	16.65
	Post-Demineralization	263	249	267	226	269	254.80	17.89
	Post-Treatment	160	193	165	146	172	167.20	17.28
	Baseline	382	377	354	379	372	372.80	11.12
	Post-Demineralization	209	193	181	213	223	203.80	16.71
	Post-Treatment	189	169	170	182	170	176.00	9.03
	Baseline	389	396	361	395	388	385.80	14.31
	Post-Demineralization	249	219	253	244	251	243.20	13.94
	Post-Treatment	206	200	216	219	231	214.40	12.01
	Baseline	407	393	376	384	376	387.20	13.10
	Post-Demineralization	216	212	213	221	216	215.60	3.51
	Post-Treatment	156	153	154	183	168	162.80	12.79
0.05% Aspartic acid	Baseline	295	319	310	288	284	299.20	14.86
	Post-Demineralization	117	126	122	85	88	107.60	19.55
	Post-Treatment	71	110	114	122	89	101.20	20.83
	Baseline	328	328	319	330	336	328.20	6.10
	Post-Demineralization	154	180	173	166	183	171.20	11.65
	Post-Treatment	148	165	126	173	167	155.80	19.07
	Baseline	308	322	346	352	326	330.80	18.03
	Post-Demineralization	180	159	141	151	166	159.40	14.81
	Post-Treatment	105	120	94	90	89	99.60	13.05
	Baseline	364	354	342	336	364	352.00	12.73
	Post-Demineralization	219	238	245	234	197	226.60	19.09

Treatment Group	Stage	HK1	HK2	HK3	HK4	HK5	Average	StnDev
	Post-Treatment	159	157	199	182	166	172.60	17.73
	Baseline	345	368	361	340	348	352.40	11.67
	Post-Demineralization	184	173	167	172	146	168.40	13.97
	Post-Treatment	141	136	150	133	144	140.80	6.69
	Baseline	351	381	340	389	376	367.40	20.89
	Post-Demineralization	184	179	164	182	199	181.60	12.50
	Post-Treatment	118	119	117	114	118	117.20	1.92
	Baseline	379	361	381	348	369	367.60	13.59
	Post-Demineralization	189	171	174	184	141	171.80	18.70
	Post-Treatment	116	104	120	127	139	121.20	12.99
	Baseline	396	371	386	351	391	379.00	18.23
	Post-Demineralization	279	250	265	301	280	275.00	18.99
	Post-Treatment	171	155	192	213	191	184.40	22.15
	Baseline	395	389	366	372	377	379.80	11.99
	Post-Demineralization	187	200	164	171	179	180.20	14.02
	Post-Treatment	51	73	54	62	58	59.60	8.56
	Baseline	435	411	404	404	402	411.20	13.74
	Post-Demineralization	232	277	244	228	240	244.20	19.40
	Post-Treatment	155	191	193	188	184	182.20	15.58
0.2% Aspartic acid	Baseline	294	300	296	299	313	300.40	7.44
	Post-Demineralization	223	222	219	220	186	214.00	15.73
	Post-Treatment	129	89	109	98	102	105.40	15.04
	Baseline	337	336	311	322	319	325.00	11.25
	Post-Demineralization	218	221	211	242	217	221.80	11.86
	Post-Treatment	74	77	83	81	93	81.60	7.27

Treatment Group	Stage	HK1	HK2	HK3	HK4	HK5	Average	StnDev
	Baseline	325	339	324	348	321	331.40	11.59
	Post-Demineralization	184	197	174	171	164	178.00	12.83
	Post-Treatment	108	112	106	114	114	110.80	3.63
	Baseline	332	368	377	345	330	350.40	21.22
	Post-Demineralization	209	219	194	201	240	212.60	17.92
	Post-Treatment	118	113	125	143	135	126.80	12.26
	Baseline	339	352	381	357	335	352.80	18.17
	Post-Demineralization	149	141	181	143	141	151.00	17.09
	Post-Treatment	53	75	54	55	67	60.80	9.76
	Baseline	384	351	369	369	361	366.80	12.13
	Post-Demineralization	209	218	218	207	209	212.20	5.36
	Post-Treatment	139	156	132	154	121	140.40	14.81
	Baseline	354	361	377	360	389	368.20	14.41
	Post-Demineralization	313	277	232	245	255	264.40	31.76
	Post-Treatment	74	68	94	58	87	76.20	14.46
	Baseline	379	393	376	374	372	378.80	8.35
	Post-Demineralization	224	201	229	220	228	220.40	11.41
	Post-Treatment	94	115	106	103	124	108.40	11.50
	Baseline	391	381	374	372	388	381.20	8.35
	Post-Demineralization	243	243	259	263	273	256.20	13.08
	Post-Treatment	124	99	117	118	130	117.60	11.63
	Baseline	393	419	382	421	395	402.00	17.18
	Post-Demineralization	180	165	210	214	223	198.40	24.68
	Post-Treatment	99	91	85	72	83	86.00	10.00
0.4% Aspartic acid	Baseline	301	307	308	283	300	299.80	10.03

Treatment Group	Stage	HK1	HK2	HK3	HK4	HK5	Average	StnDev
	Post-Demineralization	186	187	215	224	192	200.80	17.51
	Post-Treatment	79	94	71	70	73	77.40	9.91
	Baseline	308	322	319	343	335	325.40	13.76
	Post-Demineralization	177	156	192	173	226	184.80	26.36
	Post-Treatment	72	61	86	78	82	75.80	9.76
	Baseline	343	352	324	305	330	330.80	18.10
	Post-Demineralization	178	187	176	176	161	175.60	9.34
	Post-Treatment	77	65	65	53	53	62.60	10.04
	Baseline	343	360	363	326	364	351.20	16.45
	Post-Demineralization	164	160	183	174	183	172.80	10.62
	Post-Treatment	67	66	59	64	54	62.00	5.43
	Baseline	336	340	349	358	381	352.80	17.91
	Post-Demineralization	221	233	221	209	203	217.40	11.70
	Post-Treatment	66	68	61	70	70	67.00	3.74
	Baseline	336	366	377	379	376	366.80	17.94
	Post-Demineralization	191	217	201	208	219	207.20	11.58
	Post-Treatment	92	111	123	70	90	97.20	20.46
	Baseline	381	368	364	371	357	368.20	8.87
	Post-Demineralization	237	225	249	221	244	235.20	12.01
	Post-Treatment	72	89	86	70	73	78.00	8.80
	Baseline	388	388	386	369	364	379.00	11.58
	Post-Demineralization	228	260	247	244	219	239.60	16.20
	Post-Treatment	52	51	59	63	60	57.00	5.24
	Baseline	388	396	377	368	374	380.60	11.26
	Post-	165	197	196	186	184	185.60	12.90

Treatment Group	Stage	HK1	HK2	HK3	HK4	HK5	Average	StnDev
	Demineralization							
	Post-Treatment	61	60	59	69	78	65.40	8.08
	Baseline	406	391	404	400	417	403.60	9.45
	Post-Demineralization	228	227	202	225	219	220.20	10.76
	Post-Treatment	59	57	56	52	56	56.00	2.55

HK- Hardness Knoop

Table S8. Hardness Knoop average values of each enamel sample in the third experiment (assessment of remineralizing potential of aspartic acid combined with diverse calcium sources).

**Treatment Group**	**Average Baseline HK**	**Average Post-Demineralization HK**	**Average Post-Treatment HK**
**0.5% Aspartic acid + 1% Dicalcium phosphate dihydrate**	318.40	191.40	143.20
	319.40	193.40	151.20
	336.60	163.80	137.40
	336.80	169.00	122.40
	360.60	138.40	116.80
	361.60	172.40	124.40
	371.40	254.80	167.20
	372.80	203.80	176.00
	385.80	243.20	214.40
	387.20	215.60	162.80
**0.5% Aspartic acid + 1% CaMgZn**	300.40	214.00	105.40
	325.00	221.80	81.60
	331.40	178.00	110.80
	350.40	212.60	126.80
	352.80	151.00	60.80
	366.80	212.20	140.40
	368.20	264.40	76.20

Treatment Group	Average Baseline HK	Average Post-Demineralization HK	Average Post-Treatment HK
	378.80	220.40	108.40
	381.20	256.20	117.60
	402.00	198.40	86.00
**0,5% Aspartic acid + 1% nanoXim®•CarePaste Hydroxyapatite (nano)**	310.80	129.20	162.40
	324.20	198.20	220.80
	358.60	226.80	245.60
	349.80	233.60	272.00
	354.60	222.40	270.60
	366.40	282.80	334.60
	368.40	199.20	279.60
	378.60	239.60	298.00
	381.40	266.80	297.00
	401.40	241.60	294.20
**1% Aspartic acid + 1.5% TCP**	310.80	195.20	229.00
	323.60	157.20	176.20
	332.40	197.60	214.40
	349.20	199.80	213.00
	355.80	216.40	258.80
	365.40	155.20	224.60
	368.60	190.60	212.00
	377.80	207.00	216.60
	382.00	232.60	278.20
	397.20	181.80	221.00
**0,2% Aspartic acid in artificial saliva**	334.00	175.40	203.20
	336.00	177.80	213.40
	344.20	168.00	176.80
	352.00	181.40	203.60

Treatment Group	Average Baseline HK	Average Post-Demineralization HK	Average Post-Treatment HK
	352.00	201.60	244.60
	353.20	200.40	213.40
	355.20	206.80	235.80
	373.80	210.00	220.60
	375.40	218.00	258.20
	381.40	228.80	252.40
**0.05% Aspartic acid**	299.20	107.60	101.20
	328.20	171.20	155.80
	330.80	159.40	99.60
	352.00	226.60	172.60
	352.40	168.40	140.80
	367.40	181.60	117.20
	367.60	171.80	121.20
	379.00	275.00	184.40
	379.80	180.20	59.60
	411.20	244.20	182.20
**1450 ppm F (Positive Control)**	317.40	218.80	224.40
	319.40	181.00	180.80
	336.40	211.40	211.60
	337.00	230.00	238.80
	360.40	186.00	193.80
	362.00	234.60	236.40
	371.40	195.00	216.20
	373.20	194.60	204.60
	385.40	199.00	200.40
	389.20	219.60	242.20
**0.5% Aspartic acid + Monofluorophosphate**	317.00	168.40	248.40
	320.00	148.20	324.20

Treatment Group	Average Baseline HK	Average Post-Demineralization HK	Average Post-Treatment HK
	335.60	166.80	229.40
	340.40	170.40	287.00
	358.00	285.00	314.00
	363.80	184.00	216.40
	371.20	165.60	210.00
	373.80	181.80	254.20
	385.20	212.40	274.60
	392.20	244.20	302.40
**Deionized water (Negative Control)**	312.80	173.60	177.00
	321.40	158.20	161.60
	334.80	154.80	138.40
	342.80	228.60	213.00
	356.80	178.80	152.40
	364.20	180.60	162.00
	370.40	116.60	123.80
	374.20	195.20	159.20
	383.60	252.80	212.20
	399.40	215.80	180.60
**0.2% Aspartic acid**	312.80	168.20	197.80
	321.60	154.00	180.80
	334.20	185.80	220.20
	343.00	212.40	257.00
	356.60	223.00	255.20
	364.40	182.00	222.60
	370.40	185.00	251.80
	374.60	214.60	229.00
	383.20	199.00	249.80
	395.60	215.00	237.80

Treatment Group	Average Baseline HK	Average Post-Demineralization HK	Average Post-Treatment HK
**0.4% Aspartic acid**	299.80	200.80	77.40
	325.40	184.80	75.80
	330.80	175.60	62.60
	351.20	172.80	62.00
	352.80	217.40	67.00
	366.80	207.20	97.20
	368.20	235.20	78.00
	379.00	239.60	57.00
	380.60	185.60	65.40
	403.60	220.20	56.00

Table S9. Percentage of surface microhardness recovery (%SMHR) calculated for each enamel sample, average %SMHR and standard deviations (StnDev) calculated for treatment groups in the third experiment (assessment of remineralizing potential of aspartic acid combined with diverse calcium sources).

Treatment Group	% SMHR	Average % SMHR	StnDev (% SMHR)
**0.5% Aspartic acid + 1% Dicalcium phosphate dihydrate**	53.84	**45.43**	24.64
	102.44		
	37.09		
	68.59		
	39.73		
	18.02		
	21.60		
	37.71		
	36.00		
	39.32		
**0.5% Aspartic acid + 1% CaMgZn**	18.28	**33.08**	14.84
	17.94		
	14.26		

Treatment Group	% SMHR	Average % SMHR	StnDev (% SMHR)
	33.05		
	36.46		
	61.96		
	47.52		
	42.01		
	26.35		
	32.92		
**0,5% Aspartic acid + 1% nanoXim®•CarePaste Hydroxyapatite (nano)**	57.17	**26.16**	13.91
	22.99		
	19.90		
	38.90		
	26.60		
	16.55		
	23.36		
	4.77		
	23.56		
	27.80		
**1% Aspartic acid + 1.5% TCP**	20.47	**22.54**	8.65
	15.99		
	23.18		
	34.15		
	24.10		
	22.26		
	36.03		
	9.00		
	27.58		
	12.62		
**0,2% Aspartic acid in artificial saliva**	17.53	**16.21**	8.35

Treatment Group	% SMHR	Average % SMHR	StnDev (% SMHR)
	22.50		
	4.99		
	13.01		
	28.59		
	8.51		
	19.54		
	6.47		
	25.54		
	15.47		
**Aspartic acid 0.05%**	-3.34	**-35.13**	24.84
	-9.81		
	-34.89		
	-43.06		
	-15.00		
	-34.66		
	-25.84		
	-87.12		
	-60.42		
	-37.13		
**1450 ppm F (Positive Control)**	5.68	**5.15**	4.84
	-0.14		
	0.16		
	8.22		
	4.47		
	1.41		
	12.02		
	5.60		
	0.75		

Treatment Group	% SMHR	Average % SMHR	StnDev (% SMHR)
	13.33		
**0.5% Aspartic acid + Monofluorophosphate 1450 ppm**	2.44	**-11.07**	11.16
	2.08		
	-9.11		
	-13.66		
	-14.83		
	-10.13		
	2.84		
	-20.11		
	-31.04		
	-19.17		
**Deionized water (Negative Control)**	-37.95	**-29.21**	18.38
	-33.49		
	-15.28		
	-27.77		
	-9.72		
	-25.37		
	-75.13		
	-16.45		
	-20.20		
	-30.77		
**0.2% Aspartic acid**	-125.69	**-87.69**	47.76
	-135.85		
	-43.81		
	-62.26		
	-44.70		
	-46.44		
	-181.31		

Treatment Group	% SMHR	Average % SMHR	StnDev (% SMHR)
	-70.71		
	-110.88		
	-55.21		
**0.4% Aspartic acid**	-124.65	**-91.74**	27.01
	-77.52		
	-72.81		
	-62.11		
	-111.08		
	-68.92		
	-118.20		
	-130.99		
	-61.64		
	-89.53		
